# Cutaneous dysbiosis in girls with vulvar lichen sclerosus

**DOI:** 10.1128/spectrum.02674-24

**Published:** 2025-05-19

**Authors:** Liying Sun, Huihui Gao, Hongyu Chen, Chenfei Ren, Junwen Zhang, Qiuxiang Shen, Li Zhu, Dandan Chen, Lingling Jin, Chaoqun Wang, Fengxia Li, Lan Yu

**Affiliations:** 1 Department of Pediatrics and Adolescent Gynecology, Children’s Hospital, Zhejiang University School of Medicine Children's Hospital, National Clinical Research Center for Child Health 605254, Hangzhou, China; 2 Children’s Hospital, Zhejiang University School of Medicine Children's Hospital, National Clinical Research Center for Child Health 605254, Hangzhou, China; Lerner Research Institute, Cleveland, Ohio, USA

**Keywords:** prepubertal girls, vulvar lichen sclerosus, skin microbiotas, 16S rRNA, cutaneous dysbiosis

## Abstract

Vulvar lichen sclerosus (VLS) is a chronic skin condition affecting the vulva, causing significant discomfort, but its etiology in prepubertal girls remains poorly understood. VLS presents with itching, irritation, and pain. Recent studies suggest that cutaneous dysbiosis might play a role in VLS. Our study aims to investigate differences in the vulvar skin microbiota among prepubertal girls with VLS, those with labial adhesions, and healthy controls, and to explore potential microbial links to VLS. We performed a comparative analysis of 16S ribosomal RNA (rRNA) sequences from vulvar skin samples of 18 girls with VLS, 15 girls with labial adhesions, and 11 healthy girls. Microbial diversity was assessed using α diversity, β diversity, and LEfSe, and functional microbial pathways were predicted. No differences were observed in α diversity among groups. However, β diversity analysis revealed significant differences in microbial composition (Jaccard, *P* = 0.001; unweighted UniFrac, *P* = 0.01). VLS patients had increased levels of *Parvimonas* and *Fastidiosipila* and differed from controls and labial adhesion cases in specific taxa. The NAD salvage pathway was notably associated with VLS. These findings suggest that cutaneous dysbiosis may contribute to VLS pathogenesis, providing insights into the microbial changes associated with the disease. Identifying microbial dysbiosis in VLS patients offers new perspectives on its pathogenesis and potential treatment strategies.

**IMPORTANCE:**

Cutaneous dysbiosis in vulvar lichen sclerosus (VLS) may play a key role in disease pathogenesis, especially when specific microbial imbalances persist in affected patients. However, most clinical evaluations focus on symptoms rather than microbial composition, risking missed opportunities for microbiome-targeted interventions. Thus, this study highlights the importance of microbiota surveillance as a potential tool for improving the diagnosis and treatment of VLS.

## INTRODUCTION

Vulvar lichen sclerosus (VLS) is a prevalent chronic inflammatory skin lesion which is characterized by an ivory white patch on the non-hairy area of the vulva (1). The main clinical manifestations include vulva pruritus, burning, tingling, and pain, with severe cases experiencing bleeding from cleft skin, constipation, and dysuria (2). Although VLS can occur at any age, it is more frequently observed in pre-adolescent and postmenopausal individuals (3, 4). The exact prevalence of VLS is unknown, but it is estimated to be 1.7% in gynecological practice and 1 in 300–1,000 patients referred to dermatologists (5). The incidence of VLS in preadolescent girls is approximately 1 in 900 (6). If left untreated, VLS can lead to vulva atrophy, adhesion, scar formation, and loss of normal anatomy and function, affecting the quality of life and potentially increasing the risk of cancer (7).

Due to the large number of lymphocyte and plasma cell infiltration in the dermis, most scholars believe that VLS is an autoimmune disease (1, 8, 9). Exogenous factors, such as drugs or disruptions to the body’s microbiome, are speculated to potentially contribute to the development of VLS. Some infectious agents, including Borrelia burgdorferi, human papillomavirus (HPV), hepatitis C virus, and Epstein-Barr virus, have been identified in the skin or blood of patients; however, their precise role in the disease remains inconclusive (10–13). It is known that imbalanced microbial communities can cause immunity dysfunction, leading to the development of diseases (14). Recent studies have also pointed toward the possible role of the bacterial environment of skin in the pathogenesis of VLS (15, 16). A previous study on prepubescent girls with VLS found that the relative abundance of *Porphyromonas spp., Parvimonas spp., Peptoniphilus spp., Prevotella spp., Dialister spp.,* and *Peptostreptococcus spp*. was higher on the vulvar skin compared to controls, while that of *Corynebacterium spp*. was lower (15). However, this study was limited by its small sample size of five cases and three controls, and the conclusion requires further confirmation.

We thus performed a cross-sectional investigation to explore whether significant differences exist in the vulvar microbiota between girls with VLS and healthy girls or girls with other vulvar diseases.

## MATERIALS AND METHODS

### Patient and sample collection

In the present study, we recruited 18 pediatric inpatients with VLS from the Department of Pediatric and Adolescent Gynecology at Children’s Hospital, Zhejiang University School of Medicine, between December 2021 and June 2022. The diagnostic criteria of VLS included the presence of itching, burning sensations, fragile and atrophic skin lesions, fissures, erosions, intense pruritus, hyperkeratotic lesions, and ecchymoses in the affected regions. In addition, a 4 mm punch skin biopsy was collected from each patient to confirm the diagnosis of active lichen sclerosis while excluding other potential conditions such as lichen planus, psoriasis, and vulvar intraepithelial neoplasia.

As controls, we included 11 girls with Nevus of vulva (NV) and without a history of vulvoaginitis or dermatosis who had undergone hospital attendance for nevus resection and 15 girls with Labial adhesions (LA) without VLS or lichen planus history. Control participants were defined as those below 14 years of age. All the participants had not received antibiotic therapy within the last 6 months and no topical corticosteroid and calcineurin inhibitor therapies within 1 month (17–19).

The participants were examined in the lithotomy position. Disposable swabs (Yangzhou Jikang Medical Equipment Co., Ltd) were placed between the bilateral labia majora and minora, gently rotated for 5 seconds on each side. The swabs were then placed in 1 mL of RNALater solution tubes and shaken several times until the DNA was thoroughly mixed with the solution. The samples were stored at −80°C until DNA extraction was performed. The study was ethically approved by the Human Subjects Committees of Children’s Hospital, Zhejiang University School of Medicine (2022-IRB-210). All the children provided statements of assent, and legal guardians signed informed consent.

### 16S rRNA amplification and sequencing

Microbiome DNA was extracted from the skin samples using the OMEGA Soil DNA Kit (M5635-02) (Omega Bio-Tek, Norcross, GA, USA), following the manufacturer’s instructions. The V3–V4 region of bacterial 16S rRNA genes was amplified using the forward primer 338F (5′-ACTCCTACGGGAGGCAGCA-3′) and the reverse primer 806R (5′-GGACTACHVGGGTWTCTAAT-3′). Sample-specific 7 bp barcodes were incorporated into the primers for multiplex sequencing. The PCR mixture comprised 5 µL of buffer (5×), 0.25 µL of Fast pfu DNA Polymerase (5 U/µL), 2 µL (2.5 mM) of dNTPs, 1 µL (10 μM) of each Forward and Reverse primer, 1 µL of DNA Template, and 14.75 µL of ddH2O. The thermal cycling protocol consisted of an initial denaturation at 98°C for 5 min, followed by 25 cycles of denaturation at 98°C for 30 s, annealing at 53°C for 30 s, and extension at 72°C for 45 s, with a final extension at 72°C for 5 min. PCR amplicons were purified with Vazyme VAHTSTM DNA Clean Beads (Vazyme, Nanjing, China) and quantified using the Quant-iT PicoGreen dsDNA Assay Kit (Invitrogen, Carlsbad, CA, USA). After individual quantification, the amplicons were pooled in equal amounts and subjected to pair-end 2 × 250 bp sequencing on the Illumina NovaSeq platform using NovaSeq 6000 SP Reagent Kit (500 cycles) at Suzhou PANOMIX Biomedical Tech Co., LTD.

### Bioinformatic and statistical analyses

16S rRNA sequences were first assembled and screened for low quality and short length using VSEARCH v2.4.3. Next, QIIME2 (v2020.11.1) was used to process the data (20). The amplicon sequence variant (ASV) denoising was performed using DADA2, and taxonomic assignments were performed using the Greengenes2 reference database (21). Alpha diversity parameters, including observed features, Pielou, and Shannon diversity, as well as the beta diversity parameters, including the Jaccard distance and unweighted Unifrac distances, were calculated. To assess differences among the three groups, permutational multivariate analysis of variance (PERMANOVA) and Kruskal-Wallis one-way analysis of variance (Kruskal-Wallis) were conducted. The ggplots2 package in R was used for visualization.

To identify the taxa responsible for the differences among the three groups, taxa summaries generated in QIIME2 were reformatted and input into LDA effect size (LEfSe) through the Huttenhower Lab Galaxy Server (https://huttenhower.sph.harvard.edu/lefse/). This algorithm used nonparametric statistical tests to compare individual taxa between VLS and NV groups, as well as the VLS and LA groups. The abundant taxa were ranked by their linear discriminant analysis (LDA) log-scores. Differentially abundant taxa in the corresponding groups, with statistical significance at an alpha of 0.05 and LDA log-scores exceeding ±2.0, were visually displayed as bar plots.

Finally, PICRUSt2 (22) was used to predict the function of 16S rRNA sequences in each group. The MetaCyc (23) database was used to annotate the metabolic pathways and enzymes. STAMP (24) was used for analyzing differences between groups. Welch’s t-test statistical test with 95% confidence intervals was used for the comparison. A *P*-value less than 0.05 was considered statistically significant.

## RESULTS

### Demographic characteristics of the patients

A total of 18 patients with VLS, 15 patients with LA, and 11 controls with NV were included for this analysis. The basic characteristics of all individuals are summarized in Table 1, and details of each individual are presented in Table S1. The average age at recruitment in the VLS group was 7.47 ± 2.09 years with an average age of onset for this group at 6.52 ± 2.23 years. The control group had an average age of 8.38 ± 2.67 years, while the LA group exhibited a relatively younger average age of 4.51 ± 1.84 years. There was no significant difference in age at recruitment between VLS and control groups (*P* = 0.37). However, we observed a significant age difference between VLS and LA groups (*P* < 0.01, as well as a significant difference between LA and control groups (*P* < 0.01) (data not shown). The body mass index (BMI) from all groups is in the normal range. Notably, 88.9% (16 out of 18) of VLS patients had a history of allergies, 94.4% (17 out of 18) of patients had symptoms of pruritus in the vulva, 66.7% (12 out of 18) patients had vulvar hemorrhage, 16.7% (3 out of 18) reported feeling pain, and all patients exhibited whitening of the vulva.

**TABLE 1:** Characteristics of participants in this study

		Control	
	Vulvar lichen sclerosis (*N* = 18)	Nevus of vulva (*N* = 11)	Labial adhesions (*N* = 15)
Age at recruitment (years)			
Mean (SD)	7.57 (2.09)	8.38 (2.67)	4.51 (1.84)
Median [Min, Max]	7.59 [3.58, 10.7]	7.58 [4.00, 12.5]	4.16 [2.25, 7.00]
Disease duration (months)			
Mean (SD)	12.6 (13.8)	0 (0)	9.03 (10.8)
Median [Min, Max]	6.00 [0.500, 54.0]	0 [0, 0]	6.00 [0.250, 36.0]
Age of onset (years)			
Mean (SD)	6.52 (2.23)	0 (0)	3.77 (1.96)
Median [Min, Max]	6.50 [3.00, 10.2]	0 [0, 0]	3.08 [0.830, 6.75]
BMI^*a*^			
Mean (SD)	15.68 (2.10)	15.98 (2.70)	14.16 (1.85)
Median [Min, Max]	15.43 [12.31, 21.14]	15.19 [13.15, 21.49]	13.46 [12.01, 18.64]
Allergy history			
Yes	16 (88.9%)	0 (0%)	0 (0%)
No	2 (11.1%)	0 (0%)	0 (0%)
Missing	0 (0%)	11 (100%)	15 (100%)
Pruritus vulvae			
Yes	17 (94.4%)	1 (9.1%)	2 (13.3%)
No	1 (5.6%)	10 (90.9%)	13 (86.7%)
Missing	0 (0%)	0 (0%)	0 (0%)
Vulvar hemorrhage			
Yes	12 (66.7%)	0 (0%)	0 (0%)
No	6 (33.3%)	11 (100%)	15 (100%)
Missing	0 (0%)	0 (0%)	0 (0%)
Vulval pain			
Yes	3 (16.7%)	1 (9.1%)	0 (0%)
No	15 (83.3%)	10 (90.9%)	15 (100%)
Missing	0 (0%)	0 (0%)	0 (0%)
Whiten			
Yes	18 (100%)	0 (0%)	0 (0%)
No	0 (0%)	11 (100%)	15 (100%)
Missing	0 (0%)	0 (0%)	0 (0%)

^*a*^ Body mass index.

### Sequencing data analysis

**Fig 1:**
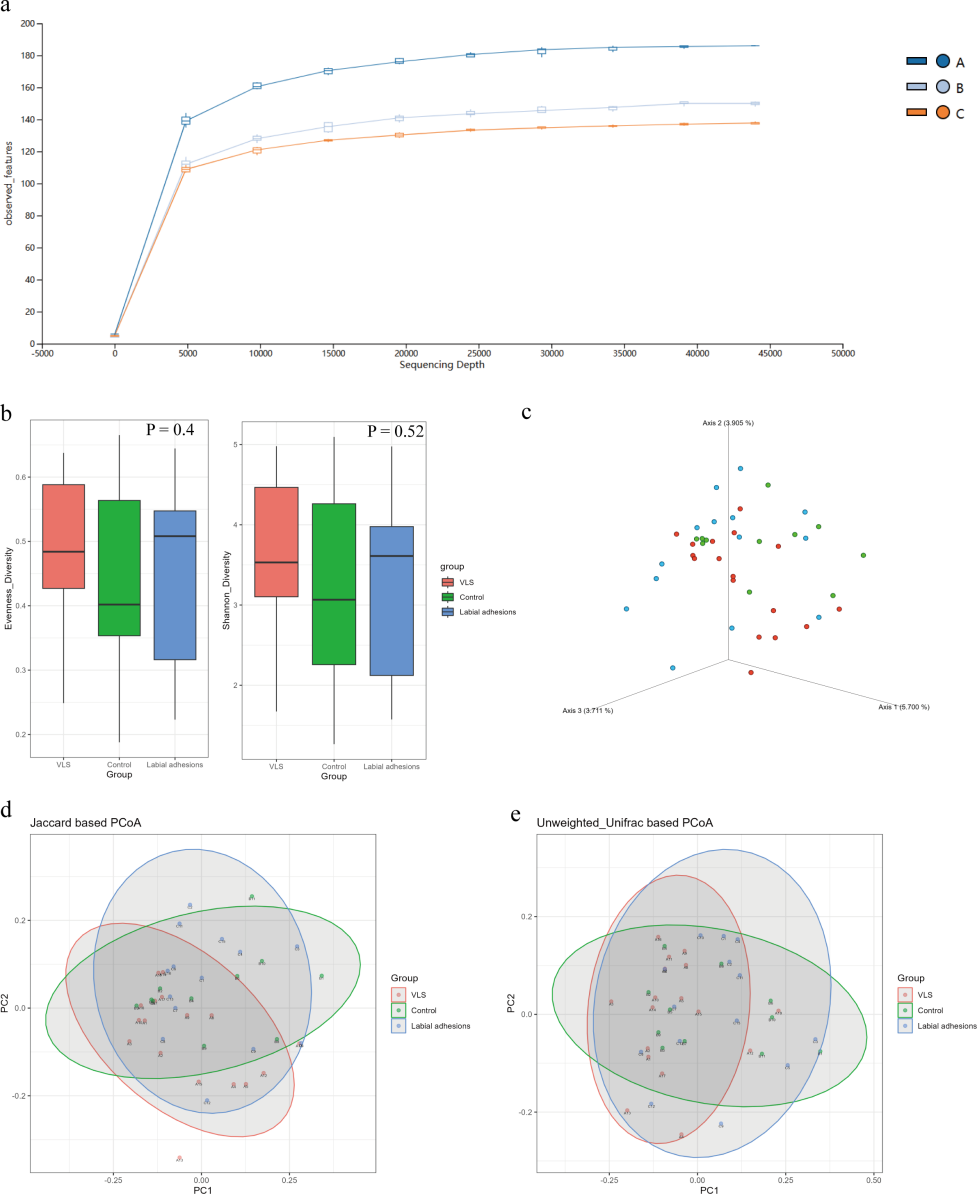
Sample quality control and microbial diversity analyses of the three groups. (**a**) Rarefaction curve of three groups (VLS group: *n* = 17; control (NV) group: *n* = 11; LA group: *n* = 14). (**b**) Alpha diversity measurements by the Shannon and Pielou diversity index. (**c, d, and e**) Beta diversity measurements by 3D-PCoA (**c**) and PCoA (**d and e**).

A total of 4,701,318 sequences were collected from the 44 samples, with an average of 106,848 sequences in each sample. After filtering, 2,961,117 high-quality sequences were used for downstream analysis. The sequence number before and after filtering in all samples is shown in Fig. S1a. We took sequence depths as the abscissa and the observed features as the ordinate to draw the rarefaction curve. As shown in Fig. 1a, the curves of all three groups tend to reach a plateau after 40,000 sequences, suggesting the sequencing depths are appropriate and sufficient to capture enough feature diversity. Furthermore, the VLS group had higher richness compared to the other two groups. We visually examined each sample in the three groups to ensure the credibility of the data under the test sequencing depth (Fig. S1b through d). Except for sample A6 and sample C14, all the other samples showed consistency. Furthermore, the observed feature index of all samples from three groups is shown in Fig. S1e, sample A6 and sample C14 were identified as outliers and removed for further analysis.

### Differences in microbial compositions at the phylum and genus levels

A total of 3,407 ASVs were obtained from all three groups. Among them, 252 ASVs were overlapped across the three groups, while 1,532 ASVs were uniquely identified in the VLS group (Fig. S2a). At the phylum level, the five most abundant bacteria were *Proteobacteria, Bacteroidetes, Firmicutes A, Firmicutes_C,* and *Actinobacteria* (Fig. S2b). Meanwhile, at the genus level, the 10 most abundant bacteria included *Peptoniphilus_E_647464, Prevotella, Porphyromonas_A_859426, Porphyromonas_A_859424, Anaerococcus, Dialister, Ezakiella, Fenollaria,* and *Peptoniphilus_A* (Fig. S2c).

Compared to asymptomatic controls, girls with VLS had an increased relative abundance of *Fusobacteria* (VLS vs. control, 0.58% vs. 0.05%, *P* = 0.0315) (Table S2) at the phylum level. Girls with VLS had an increased relative abundance of *Firmicutes_B_370539* (VLS vs. LA, 0.06% vs. 0.01%, *P* = 0.0036) (Table S2) at the phylum level. No significant difference was found between the LA and control groups. At the genus level, *Parvimonas* and *Fastidiosipila* increased significantly in the VLS group compared to the control group (Table 2, both adjusted *P* < 0.05). In addition, *Fusobacterium_C* and *Peptostreptococcus* showed a trend toward increased abundance in the VLS group, although no statistical significance was observed between these two groups. Furthermore, a significant increase in *Peptostreptococcus* was also observed in the LA group compared to the NV control group. When comparing the VLS and LA groups, a higher relative abundance of *Anaerococcus, Parvimonas*, and *Fastidiosipila Moryella* spp., *Gallicola* spp., and *Peptococcus* was observed in the VLS group (Table 2, all adjusted *P* < 0.05). Conversely, *Varibaculum* was significantly increased in the LA group compared to both the VLS and NV control groups, suggesting that the abundance of *Varibaculum* may serve as a potential biomarker to distinguish LA from other diseases or control groups.

**TABLE 2:** The average relative abundance of microbiota at the genus level in the VLS group and control groups^*a*^

	VLS (%）	NV (%）	LA (%)	p1	p2	p3
*g__Pseudomonas_E_647464*	49.53	52.60	48.85	0.7279	0.7279	0.7279
*g__Prevotella*	11.98	12.03	20.85	0.6995	0.0837	0.1247
*g__Porphyromonas_A_859426*	7.09	7.55	1.87	0.6727	0.0585	0.0585
*g__Fenollaria*	4.96	5.35	6.39	0.6746	0.67471	0.6747
*g__Porphyromonas_A_859424*	4.38	1.89	2.32	0.2289	0.2394	0.5878
*g__Dialister*	3.35	3.38	4.57	0.7567	0.4128	0.6264
*g__Ezakiella*	1.86	1.74	2.24	0.8903	0.8708	0.8708
*g__Corynebacterium*	1.67	0.57	0.42	0.4356	0.4356	0.4356
*g__Peptoniphilus_A*	1.51	1.47	1.65	0.6576	0.4173	0.6576
*g__Anaerococcus*	1.47	1.07	0.90	0.4247	0.0462**^*b*^**	0.4258
*g__Campylobacter_B*	1.17	1.00	0.83	0.9034	0.9034	0.9034
*g__Methylobacterium*	0.90	0.89	0.63	0.6384	0.3306	0.3306
*g__Peptoniphilus_B_226777*	0.86	0.49	0.74	0.5386	0.9285	0.5386
*g__Parvimonas*	0.80	0.11	0.08	0.0003^*b*^	0.0003^*b*^	0.7917
*g__Peptoniphilus_C*	0.69	0.33	0.38	0.1461	0.1461	0.6286
*g__Finegoldia*	0.55	0.73	0.60	0.5394	0.5394	0.6198
*g__Mobiluncus*	0.48	0.23	1.35	0.2997	0.2997	0.2997
*g__Fusobacterium_C*	0.45	0.02	0.14	0.0549	0.1502	0.1502
*g__Facklamia_A_322655*	0.42	0.19	0.09	0.4996	0.1692	0.4996
*g__Negativicoccus*	0.31	0.30	0.42	0.8993	0.8993	0.8993
*g__W5053*	0.29	0.20	0.21	0.9095	0.9095	0.9095
*g__Lawsonella*	0.29	0.34	0.38	0.7164	0.7164	0.8079
*g__Winkia*	0.28	0.16	0.05	0.7446	0.6416	0.6416
*g__Urinicoccus*	0.25	0.19	0.16	0.7350	0.7350	0.7350
*g__Varibaculum*	0.25	0.15	0.92	0.4975	0.0267^*b*^	0.0267^*b*^
*g__Fastidiosipila*	0.21	0.04	0.03	0.0210^*b*^	0.0174^*b*^	0.6785
*g__Helcococcus*	0.20	0.03	0.01	0.1370	0.1370	0.4281
*g__Pauljensenia*	0.20	0.23	0.09	0.6190	0.2730	0.2934
*g__KA00134*	0.19	0.17	0.04	0.9762	0.1104	0.2726
*g__Peptostreptococcus*	0.17	0.03	0.17	0.0666	0.7336	0.0378^*b*^
*g__S5.A14a*	0.16	0.06	0.18	0.3887	0.7055	0.2682
*g__Streptococcus*	0.15	0.17	0.10	0.8379	0.8379	0.8379
*g__Gleimia*	0.12	0.01	0.00	0.2289	0.2289	0.3485
*g__Sneathia*	0.11	0.03	0.01	0.4500	0.3921	0.4500
*g__Staphylococcus*	0.10	0.07	0.02	0.7114	0.3318	0.3521
*g__UBA1822*	0.10	0.10	0.24	0.8450	0.2082	0.2082
*Others*	2.49	6.09	2.08	NA	NA	NA

^*a*^ VLS, Vulvar lichen sclerosis; NV, Nevus of vulva; LA, Labial adhesions; NA, not applicable. p1, p2, and p3 were the results of Welch's t-test of VLS&NV, VLS&LA, NV&LA, respectively.

^*b*^ Adjusted *P* < 0.05 with Benjamini-Hochberg correction. Others include the taxa that cannot be classified and taxa with the relative abundance of less than 0.5%.

### Microbial diversity analysis

The alpha diversity, which assesses the richness and evenness of the microbiome, revealed no significant differences among the three groups based on Pielou or Shannon indices (Fig. 1b). However, beta diversity analysis using principal coordinate analysis (PCoA) suggested significant compositional differences among the three groups. The PCoA plots based on Jaccard (PERMANOVA *P* = 0.001) and Unweighted_Unifrac (PERMANOVA *P* = 0.01) distances are shown in Fig. 1d and e, respectively. In addition, three-dimensional PCoAs (3D-PCoAs) based on Jaccard distances (PERMANOVA *P* = 0.001) for the three groups are shown in Fig. 1c. Considering the age difference among the three groups, we further subdivided the individuals into different age groups. No significant differences in diversity were found among these three groups when analyzed separately (Table S3).

**Fig 2:**
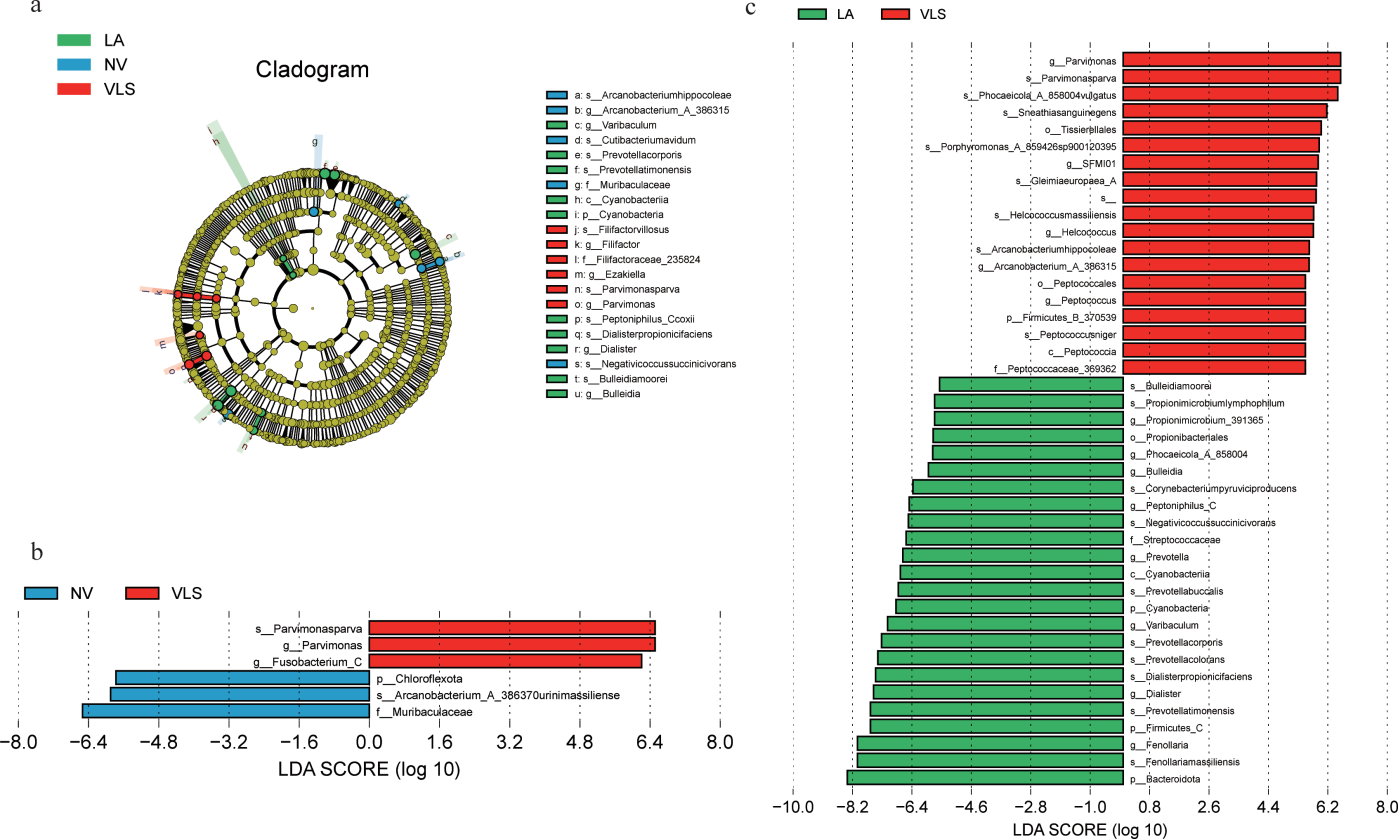
The LEfSe results of the three groups. (**a**) Taxonomic branch diagram of significant microbial species (LDA threshold of 2). (**b**) Results of the VLS group and the control (NV) group. (**c**) Results of the VLS group and the LA group.

We also used the LEfSe algorithm to identify the specific taxa with variable distributions among the groups. A total of 21 taxa were identified (Fig. 2a). Among these, six taxa were found to be over-represented in the VLS groups: *g_Parvimonas, s_Parvimonasparva, f_Filifactoraceae_235824, g_Filifactor, s_Filifactorvillosus,* and *g_Ezakiella.* 10 taxa were found to be over-represented in the LA groups, with their LDA scores from high to low: *g_Dialister, s_Dialisterinvisus, s_Prevotellatimonensis, s_Prevotellacorporis, s_Peptoniphilus_Ccoxii, g_Varibaculum, s_Bulleidiamoorei, g_Bulleidia, c_Cyanobacteriia,* and *p_Cyanobacteria*. On the other hand, five taxa were found to be over-represented in the NV control groups, with their LDA scores ranked from high to low: *s_Negativicoccussuccinicivorans, f_Muribaculaceae, s_Arcanobacteriumhippocoleae, g_Arcanobacterium_A_386315,* and *s_Cutibacteriumnamnetense.* Furthermore, when compared to control girls with NV, a statistically significantly higher relative abundance of *g_Parvimonas, s_Parvimonasparva*, and *g_Fusobacterium* was observed in girls with VLS (Fig. 2b), while the relative abundance of *p_Chloroflexota, s_Arcanobacterium_A_386370urinimassiliense,* and *f_Muribaculaceae* was significantly higher in NV subjects. Similarly, when comparing girls with LA, a significantly higher relative abundance of taxa such as *g_Parvimonas, s_Parvimonasparva, s_Phocaeicola_A_858004vulgatus,* and *s_Sneathiasanguinegens* was observed in girls with VLS. Conversely, the abundances of taxa such as *p_Bacteroidota, s_Fenollariamassiliensis, g-Fenollaria,* and *p_Firmicutes_C* were higher in the LA group (Fig. 2c).

### Predicted functional microbial pathways

**Fig 3:**
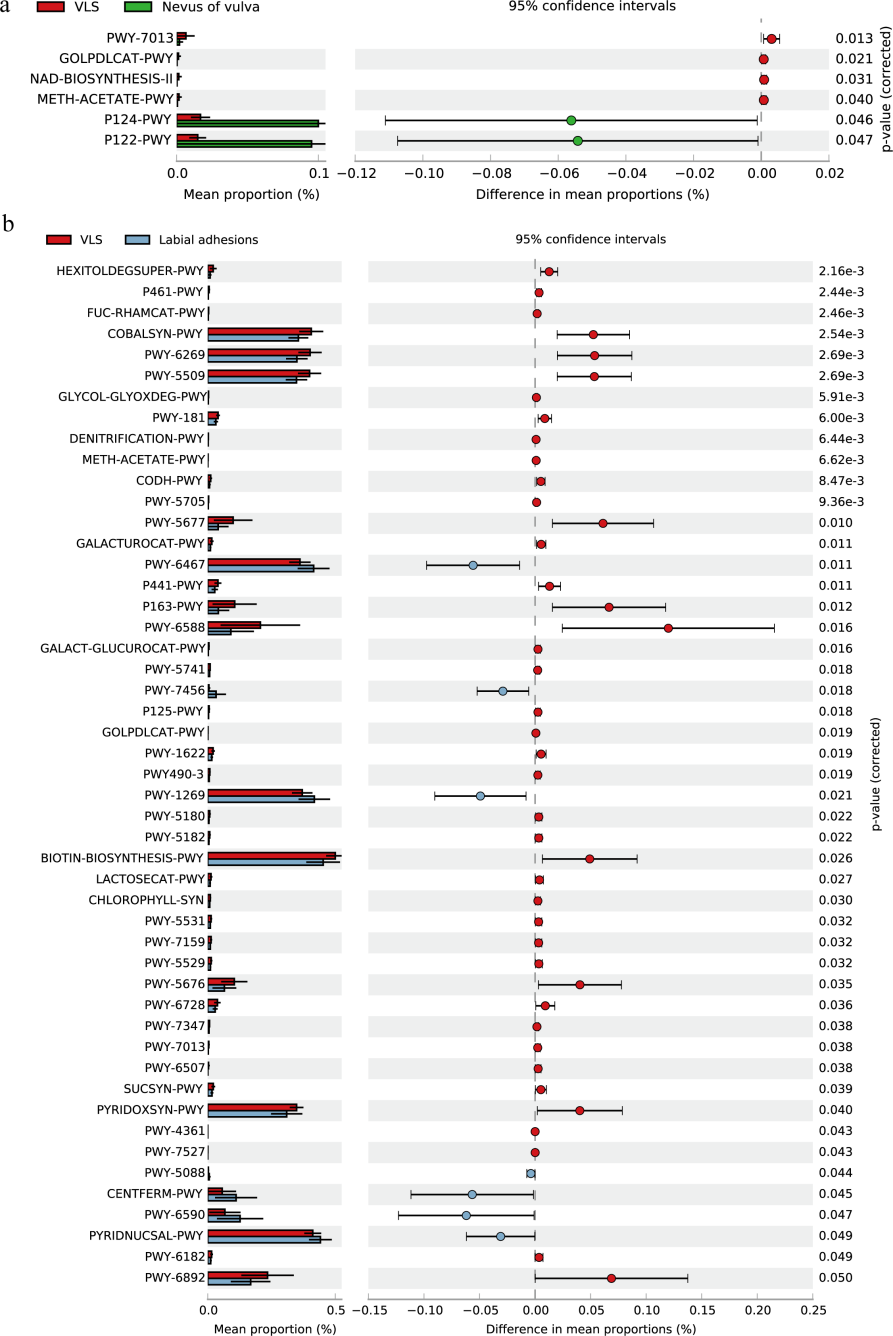
Functionally predicted MetaCyc pathways differing in proportions between the VLS group and the other two groups. (**a**) Pathways with significant differences between the VLS group and the control (NV) group. (**b**) Pathways with significant differences between the VLS group and the LA group. The bar plot shows mean proportions of differential MetaCyc pathways predicted using PICRUSt2.

A total of 393 different predicted functional pathways were identified based on the Metacyc database. Compared to the control group, L-1,2-propanediol degradation, superpathway of glycerol degradation to 1,3-propanediol, NAD salvage pathway II, and methanogenesis from acetate pathways were significantly enriched in VLS patients, while Bifidobacterium shunt and heterolactic fermentation were increased in the control group (all *P* < 0.05; Table S4; Fig. 3a). Furthermore, when comparing the VLS to the LA group, 49 significantly different pathways were highlighted (Table S5). Among them, pathways related to methanogenesis from acetate and the superpathway of glycerol degradation to 1,3-propanediol were also observed to be different between VLS and controls (Table S5; Fig. 3b).

## DISCUSSION

In this study, we observed that the mean age of onset of VLS in girls is 6.52 years, while the mean age at diagnosis is 7.57 years. This finding aligns with previous research indicating a delay in the accurate diagnosis of VLS in girls (25), suggesting a precise and timely diagnosis of VLS is still a challenge. Of note, 88.9% of girls with VLS have a history of allergies, further supporting the involvement of immunopathogenesis in this disease (9).

There were no differences in the richness and evenness of microbial communities among all three groups. However, significant differences in microbial compositions were observed among the VLS, LA, and control groups. A similar pattern of taxa composition was found in skin samples of girls with VLS (15). While in adults, both the richness and the composition of the microbiota differed between VLS and control groups (16), suggesting the pathogenesis of VLS in girls and adults may slightly differ, with cutaneous dysbiosis more frequently observed in adults.

We found that *Parvimonas* and *Fastidiosipila* were more enriched on the skin of the VLS group compared to controls. *Parvimonas* was consistently identified in the previous study as well (15). Furthermore, *Parvimonas* and *Fastidiosipila* were with higher abundance in the girls with VLS compared to the LA group, while these two taxa showed no difference between the LA and NV control group, suggesting the possibility of specific bacteria patterns to discriminate VLS against other vaginal inflammation. *Parvimonas* was found to be correlated with the clinical severity of hidradenitis suppurativa, which is considered to be an inflammatory disease (26). It has also been predominantly enriched in patients with chronic periodontitis, where its presence may contribute to the alteration of permeability and promotion of periodontitis through harmful factors and released peptides and proteins (27, 28). *Fastidiosipila* is a Gram-positive anaerobic coccus that primarily colonizes skin, oral cavity, upper airway mucosa, gastrointestinal tract, and female genitourinary tract (29). It was reported to be a biomarker for human papillomavirus infection (30). In addition, it is increased in women with cervical preneoplasia (31) and adenomyosis (32). As an autoimmune disease, VLS is often associated with an increased risk of squamous cell carcinoma (3). The higher abundance of *Parvimonas* and *Fastidiosipila* in VLS may offer valuable clinical insights, serving as potential biomarkers for diagnosis, risk assessment for squamous cell carcinoma, and monitoring disease progression. In addition, targeting these bacteria through antimicrobial therapies or microbiome-based treatments could provide new therapeutic avenues, enabling personalized care and adjunctive therapies for VLS patients. We did not find differences in *Peptostreptococcus, Porphyromonas spp., Peptoniphilus spp., Prevotella spp., Dialister spp.,* and *Corynebacterium spp.* between VLS and control groups, which were indicated in the previous study (15). The relatively small sample size of five VLS cases and three healthy controls in the previous study might limit the power of the analysis. Differences in microbial richness across racial groups may contribute to these varied outcomes (31). Furthermore, we used AVS instead of operational taxonomic unit (OTU) in our study. AVS identifies exact sequence variants rather than grouping them into clusters based on similarity, offering higher resolution than OTUs and enabling more precise detection of differences between groups. In addition, we updated the annotation database, which may lead to more comprehensive microbial identification and the reclassification of previously annotated bacterial species.

Although no significant difference was observed between VLS and controls, *Peptostreptococcus* exhibited a trend toward increased abundance in both VLS and LA groups compared to controls. In a study investigating immune biomarker expression in patients with idiopathic infertility, the presence of *Peptostreptococcus* and HPV in the endometrium correlated with the decreased expression of endometrial TGFβ1 and bFGF2 and increased expression of DEFa1 (33), suggesting its potential role in cytokine regulation. *Peptostreptococcus* in periodontitis can stimulate various immune cells and produce TNF-α, IL-6, or IL-1β to trigger an inflammatory response (34), promoting bone resorption (35) and causing irreversible destruction of the periodontium (36). The possibility of vaginal atrophy and scar formation in VLS patients may be a result of alterations in innate immune response and barrier properties (37), which are probably regulated by microbiota (38). *Fusobacterium also showed an increase in the VLS group. Fusobacterium* is enriched in tumor tissues and can promote tumor growth and metastatic progression by recruiting tumor-infiltrating immune cells (39, 40). Notably, VLS patients often exhibit an association with squamous cell carcinoma (3). The increased abundance of *Fusobacterium* in VLS patients may contribute to tumorigenesis.

The dysbiosis observed in VLS was predicted to be associated with several functional metabolic pathways, including the nicotinamide adenine dinucleotide (NAD) salvage. NAD serves as a cofactor for many metabolic reactions across cell types and exhibits anti-inflammatory effects, anti-oxidant, and barrier repair properties in inflammatory skin diseases (41). Increased NAD content has been noted in psoriatic lesions, where the NAD salvage pathway contributed to the pathogenesis by amplifying epithelial auto-inflammatory responses (42). Similar NAD metabolism patterns may also be involved in VLS pathogenesis. Therefore, further investigations into NAD levels and associated cytokines in the skin of VLS patients are necessary to elucidate their potential role. In addition, the pathway related to methanogenesis from acetate was found to be increased in the VLS group. Interestingly, previous studies suggested that treatment with ozonides combined with vitamin E acetate yields effects similar to steroid topical treatment in LS (43). This raises the possibility of alternative treatments to corticosteroids for children affected by VLS, which could be explored further by investigating the relationship between the skin microbiota and host metabolism.

Our study has several limitations. First, the sample size remains limited. However, the consistency in observed features across all included samples and minimal differences within each group indicate that these samples can be considered representative. While our results provide valuable insight, expanding the study with a larger population from multiple centers could validate and enhance our findings. Second, it is important to investigate other infection agents, such as viruses or fungi, which may also contribute to the development of VLS. Moreover, given the common occurrence of constipation in VLS patients, exploring the skin and gut microbiotas could provide valuable insights into shared or distinct mechanisms influencing VLS. Finally, we anticipated an association between observed dysbiosis and host metabolism. Further investigation into the intricate interaction between the host and potentially correlated bacteria requires a more comprehensive analysis of the microbiome and metabolome.

### Conclusion

Our study provides compelling evidence of significant alterations in the cutaneous microbiota of girls with VLS. Specifically, we observed higher levels of *Parvimonas* and *Fastidiosipila* in girls with VLS compared to the control group. These findings suggest a potential association between cutaneous dysbiosis and VLS pathogenesis. Investigating the roles of these specific bacteria may offer insights into the development of novel therapeutics.

## Data Availability

The raw sequence data reported in this paper have been deposited in the Genome Sequence Archive (44) in the National Genomics Data Center (45), China National Center for Bioinformation/Beijing Institute of Genomics, and Chinese Academy of Sciences (GSA: CRA015702) which are publicly accessible at https://ngdc.cncb.ac.cn/gsa.

## SUPPLEMENTAL MATERIAL

Supplemental figures and tables spectrum.02674-24-s0001.pdf Figures S1 and S2, and Tables S1 to S5.

ASM does not own the copyrights to Supplemental Material that may be linked to, or accessed through, an article. The authors have granted ASM a non-exclusive, world-wide license to publish the Supplemental Material files. Please contact the corresponding author directly for reuse.
