# Supplementary material for: Cutaneous dysbiosis in girls with vulvar lichen sclerosus
Source: Microbiol Spectr. 2025 May 19;13(7):e02674-24. doi: 10.1128/spectrum.02674-24 (PMC12210941; doi:10.1128/spectrum.02674-24)
Supplement: Supplemental figures and tables — Figures S1 and S2, and Tables S1 to S5. [file spectrum.02674-24-s0001.pdf]

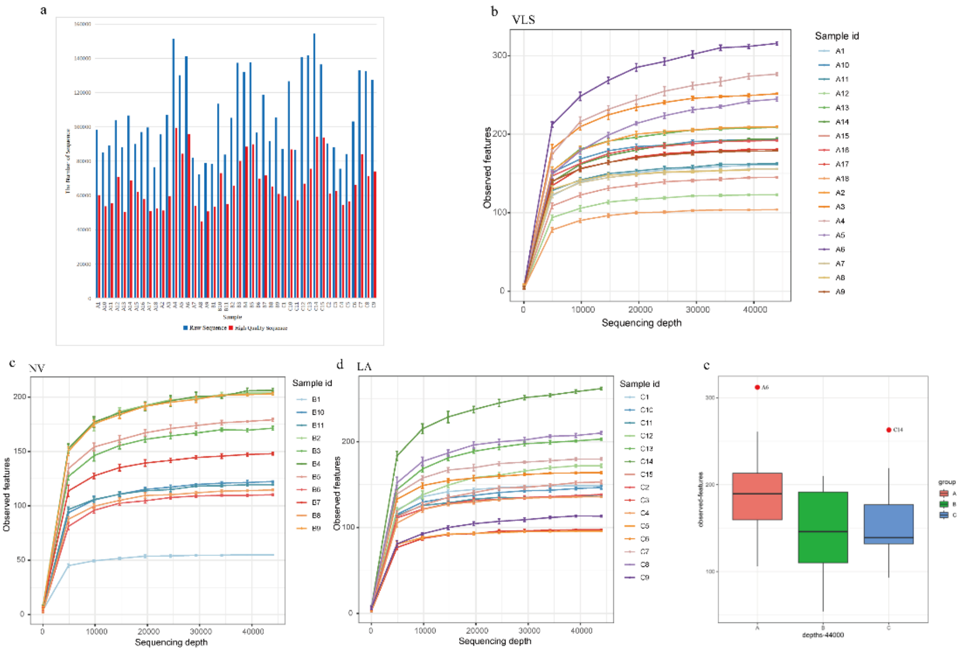

Figure S1. (a) The number of raw sequence and high quality sequence of 44 samples. (b-d) The rarefaction curve of observed features of 42 samples (b: individuals from VLS group; c: individuals from control of NV group; d: individuals from LA group). (e) The number of features among the three groups (VLS group: n=18; Control (NV) group: n=11; LA group: n=15) at the 44,000 sequence depth. A6 and C14 are outliers and excluded for further analysis.

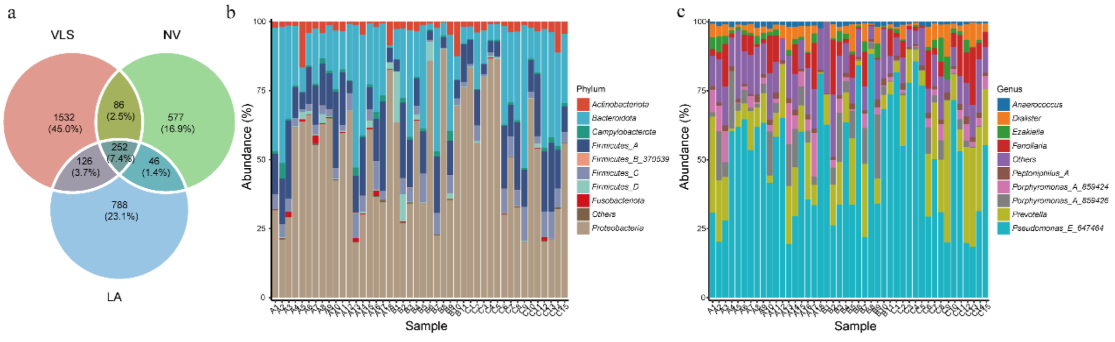

Figure S2. Venn and species composition. (a) Venn of the three groups. (b) Microbial composition at the phylum level. (c) Microbial composition at the genus level.

Table S1. The detailed characteristics of each participant

group and LA group.	group	disease	Age at recruitment	Age of onset	BMI	Pruritus vulvae	Vulvar hemorrhage	Vulval pain	Whiten	Disease duration	Allergy history
A1	A	Vulvar lichen sclerosus	4.58	4.06	15.86	yes	yes	yes	yes	6	yes
A2	A	Vulvar lichen sclerosus	5.16	4.16	15.15	yes	yes	no	yes	12	yes
A3	A	Vulvar lichen sclerosus	5.75	5.25	12.31	yes	yes	no	yes	6	yes
A4	A	Vulvar lichen sclerosus	7.67	7.67	17.02	no	yes	no	yes	0.5	no
A5	A	Vulvar lichen sclerosus	10.67	10.17	16.70	yes	yes	yes	yes	6	yes
A6	A	Vulvar lichen sclerosus	9.25	8.75	15.53	yes	yes	no	yes	6	yes
A7	A	Vulvar lichen sclerosus	10.33	10.08	16.17	yes	yes	no	yes	3	yes
A8	A	Vulvar lichen sclerosus	6.42	5.42	15.10	yes	no	no	yes	12	yes
A9	A	Vulvar lichen sclerosus	9.16	7.16	12.82	yes	yes	yes	yes	24	yes
A10	A	Vulvar lichen sclerosus	8.16	8	14.88	yes	no	no	yes	2	yes
A11	A	Vulvar lichen sclerosus	5.75	5.25	15.55	yes	yes	no	yes	6	yes
A12	A	Vulvar lichen	6.83	5.83	18.44	yes	yes	no	yes	12	yes

		sclerosus									
A13	A	Vulvar lichen sclerosus	7.5	3	21.14	yes	no	no	yes	54	yes
A14	A	Vulvar lichen sclerosus	3.58	3.08	13.37	yes	no	no	yes	6	yes
A15	A	Vulvar lichen sclerosus	10.41	7.41	17.78	yes	yes	no	yes	36	yes
A16	A	Vulvar lichen sclerosus	8.16	7.66	15.32	yes	yes	no	yes	6	no
A17	A	Vulvar lichen sclerosus	9.41	8.91	15.32	yes	no	no	yes	6	yes
A18	A	Vulvar lichen sclerosus	7.41	5.41	13.76	yes	no	no	yes	24	yes
B1	B	Control(Nevus of vulva)	12.5	12.5	15.19	no	no	no	no	0	
B2	B	Control(Nevus of vulva)	11.16	11.16	16.57	no	no	no	no	0	
B3	B	Control(Nevus of vulva)	6.66	6.66	17.98	no	no	no	no	0	
B4	B	Control(Nevus of vulva)	7.58	7.58	13.44	no	no	no	no	0	
B5	B	Control(Nevus of vulva)	6.83	6.83	19.41	no	no	no	no	0	
B6	B	Control(Nevus of vulva)	11	11	21.49	no	no	no	no	0	
B7	B	Control(Nevus of	4	4	16.29	no	no	no	no	0	

		vulva)								
B8	B	Control(Nevus of vulva)	7	7	14.30	yes	no	no	no	0
B9	B	Control(Nevus of vulva)	9.41	9.41	13.39	no	no	no	no	0
B10	B	Control(Nevus of vulva)	10.41	10.41	14.59	no	no	no	no	0
B11	B	Control(Nevus of vulva)	5.66	5.66	13.15	no	no	yes	no	0
C1	C	Labial adhesions	3	2	14.65	no	no	no	no	12
C2	C	Labial adhesions	6.58	4.58	18.64	no	no	no	no	24
C3	C	Labial adhesions	3.08	3.08	15.56	yes	no	no	no	0.25
C4	C	Labial adhesions	4.16	3.66	12.01	no	no	no	no	6
C5	C	Labial adhesions	2.83	0.83	13.50	no	no	no	no	24
C6	C	Labial adhesions	7	6.5	15.50	no	no	no	no	6
C7	C	Labial adhesions	6.5	6.5	17.36	no	no	no	no	0.5
C8	C	Labial adhesions	2.25	2.25	13.26	no	no	no	no	0.5
C9	C	Labial adhesions	6.75	6.75	12.81	no	no	no	no	0.25
C10	C	Labial adhesions	2.66	1.66	12.75	no	no	no	no	12
C11	C	Labial adhesions	3.25	3	13.46	no	no	no	no	3
C12	C	Labial adhesions	2.33	2	13.02	no	no	no	no	4
C13	C	Labial adhesions	5	4.5	13.15	no	no	no	no	6
C14	C	Labial adhesions	6.25	6.17	13.64	no	no	no	no	1
C15	C	Labial adhesions	6.08	3.08	13.15	yes	no	no	no	36

Table S2. The average relative abundance of microbiota at phylum level in the VLS group and control groups

	VLS（%）	NV（%）	LA(%)	p1	p2	p3
d__Bacteria; p__Proteobacteria	50.78	53.64	50.32	0.7208	0.7208	0.7678
d__Bacteria; p__Bacteroidota	23.73	21.94	25.10	0.8921	0.8921	0.8921
d__Bacteria; p__Firmicutes_A	14.88	12.43	14.19	0.7794	0.7794	0.7794
d__Bacteria; p__Firmicutes_C	4.05	4.27	5.40	0.6166	0.3888	0.6166
d__Bacteria; p__Actinobacteriota	3.64	3.26	3.53	0.9869	0.9869	0.9869
d__Bacteria; p__Campylobacterota	1.19	1.00	0.83	0.8707	0.8707	0.8707
d__Bacteria; p__Firmicutes_D	0.89	3.21	0.32	0.1232	0.1059	0.1059
d__Bacteria; p__Fusobacteriota	0.58	0.05	0.15	**0.0315***	0.0882	0.2593
d__Bacteria; p__Firmicutes_B_370539	0.06	0.02	0.01	0.1596	**0.0036***	0.4665
others	0.21	0.17	0.14	NA	NA	NA

Note: VLS, Vulvar lichen sclerosis; NV, Nevus of vulva; LA, Labial adhesions; NA, not applicable

P1, p2, p3 were the results of welch's t-test of VLS&NV, VLS&LA, NV&LA, respectively. * With Black font: $P<0.05$

Others include the taxa that cannon be classified and taxa with relative abundance of less than 0.5%.

Table S3. The diversity analysis based on age

**Age 0~6**	**shannon***	**pielou***	**jaccard***	**unweighted***
VLS & control (NV)	0.57	0.85	0.662	0.431
control (NV) & LA	0.34	0.20	0.101	0.317
VLS & LA	0.23	0.23	0.039	0.107
VLS & Control & LA	0.38	0.31	0.054	0.161
**Age 7~9 #**	**shannon**	**pielou**	**jaccard**	**unweighted**
VLS & control (NV)	0.31	0.31	0.727	0.909
**Age 10+#**	**shannon**	**pielou**	**jaccard**	**unweighted**
VLS & control (NV)	0.29	0.29	0.215	0.167

Note: * P value of each analysis. # not available in LA group

Table S4. The predicted metabolic pathways and the results of statistical tests between VLS and control (NV)group

Pathway	Description	p-values(Welch's t-test)
PWY-7013	L-1,2-propanediol degradation	0.0130
**GOLPDLCAT-PWY**	**superpathway of glycerol degradation to 1,3-propanediol**	**0.0206***
NAD-BIOSYNTHESIS-II	NAD salvage pathway II	0.0313
**METH-ACETATE-PWY**	**methanogenesis from acetate**	**0.0402***
P124-PWY	Bifidobacterium shunt	0.0461
P122-PWY	heterolactic fermentation	0.0468
CODH-PWY	reductive acetyl coenzyme A pathway	0.0533
KDO-NAGLIPASYN-PWY	superpathway of (Kdo)2-lipid A biosynthesis	0.0627
PWY-5837	1,4-dihydroxy-2-naphthoate biosynthesis I	0.0732
PWY-5863	superpathway of phylloquinol biosynthesis	0.0732
PWY0-1479	tRNA processing	0.0741
PWY0-1296	purine ribonucleosides degradation	0.0742
PWY-5897	superpathway of menaquinol-11 biosynthesis	0.0778
PWY-5898	superpathway of menaquinol-12 biosynthesis	0.0778
PWY-5899	superpathway of menaquinol-13 biosynthesis	0.0778
PWY-5861	superpathway of demethylmenaquinol-8 biosynthesis	0.0782
PWY-5838	superpathway of menaquinol-8 biosynthesis I	0.0786
PWY-5840	superpathway of menaquinol-7 biosynthesis	0.0791
PWY-6572	chondroitin sulfate degradation I (bacterial)	0.0840
PWY-6891	thiazole biosynthesis II (Bacillus)	0.0874
PWY-6895	superpathway of thiamin diphosphate biosynthesis II	0.0923
P125-PWY	superpathway of (R,R)-butanediol biosynthesis	0.1052

PWY-5088	L-glutamate degradation VIII (to propanoate)	0.1108
P161-PWY	acetylene degradation	0.1122
PWY0-166	superpathway of pyrimidine deoxyribonucleotides de novo biosynthesis (E. coli)	0.1138
PWY-6731	starch degradation III	0.1147
PWY0-781	aspartate superpathway	0.1203
PWY-6562	norspermidine biosynthesis	0.1233
PWY-5415	catechol degradation I (meta-cleavage pathway)	0.1314
PWY-5910	superpathway of geranylgeranyldiphosphate biosynthesis I (via mevalonate)	0.1318
PWY-922	mevalonate pathway I	0.1322
OANTIGEN-PWY	O-antigen building blocks biosynthesis (E. coli)	0.1378
PWY-5667	CDP-diacylglycerol biosynthesis I	0.1394
PWY0-1319	CDP-diacylglycerol biosynthesis II	0.1394
PWY-5178	toluene degradation IV (aerobic) (via catechol)	0.1398
P101-PWY	ectoine biosynthesis	0.1403
PWY0-162	superpathway of pyrimidine ribonucleotides de novo biosynthesis	0.1406
PHOSLIPSYN-PWY	superpathway of phospholipid biosynthesis I (bacteria)	0.1515
DENITRIFICATION-PWY	nitrate reduction I (denitrification)	0.1521
PWY-5005	biotin biosynthesis II	0.1540
PWY-5971	palmitate biosynthesis II (bacteria and plants)	0.1723
PWY-6749	CMP-legionaminate biosynthesis I	0.1753
PWY-7315	dTDP-N-acetylthomosamine biosynthesis	0.1774
FUCCAT-PWY	fucose degradation	0.1852
PWY-6713	L-rhamnose degradation II	0.1881
GLYCOL-GLYOXDEG-PWY	superpathway of glycol metabolism and degradation	0.1959
PWY0-1586	peptidoglycan maturation (meso-diaminopimelate containing)	0.1974
PWY-6210	2-aminophenol degradation	0.2004

PWY-6892	thiazole biosynthesis I (E. coli)	0.2054
PRPP-PWY	superpathway of histidine, purine, and pyrimidine biosynthesis	0.2069
PWY-6353	purine nucleotides degradation II (aerobic)	0.2128
PWY-6505	L-tryptophan degradation XII (Geobacillus)	0.2135
PWY-6944	androstenedione degradation	0.2137
PWY0-845	superpathway of pyridoxal 5'-phosphate biosynthesis and salvage	0.2141
CRNFORCAT-PWY	creatinine degradation I	0.2145
UDPNAGSYN-PWY	UDP-N-acetyl-D-glucosamine biosynthesis I	0.2151
PWY-5920	superpathway of heme biosynthesis from glycine	0.2248
COLANSYN-PWY	colanic acid building blocks biosynthesis	0.2271
PWY-6383	mono-trans, poly-cis decaprenyl phosphate biosynthesis	0.2287
PWY-6317	galactose degradation I (Leloir pathway)	0.2309
P461-PWY	hexitol fermentation to lactate, formate, ethanol and acetate	0.2311
PWY0-1277	3-phenylpropanoate and 3-(3-hydroxyphenyl)propanoate degradation	0.2357
SALVADEHYPOX-PWY	adenosine nucleotides degradation II	0.2393
PWY-3661	glycine betaine degradation I	0.2440
PWY1G-0	mycothiol biosynthesis	0.2508
PWY-6397	mycolyl-arabinogalactan-peptidoglycan complex biosynthesis	0.2525
PWY-6125	superpathway of guanosine nucleotides de novo biosynthesis II	0.2533
THISYN-PWY	superpathway of thiamin diphosphate biosynthesis I	0.2533
KETOGLUCONMET-PWY	ketogluconate metabolism	0.2686
GLCMANNANAUT-PWY	superpathway of N-acetylglucosamine, N-acetylmannosamine and N-acetylneuraminate degradation	0.2693
PWY0-1297	superpathway of purine deoxyribonucleosides degradation	0.2772
COMPLETE-ARO-PWY	superpathway of aromatic amino acid biosynthesis	0.2832
METHGLYUT-PWY	superpathway of methylglyoxal degradation	0.2856

PWY-5419	catechol degradation to 2-oxopent-4-enoate II	0.2862
FERMENTATION-PWY	mixed acid fermentation	0.2870
P105-PWY	TCA cycle IV (2-oxoglutarate decarboxylase)	0.2885
PWY-6143	CMP-pseudaminate biosynthesis	0.2906
PWY-5104	L-isoleucine biosynthesis IV	0.2929
ARO-PWY	chorismate biosynthesis I	0.2947
P164-PWY	purine nucleobases degradation I (anaerobic)	0.2951
ENTBACSYN-PWY	enterobactin biosynthesis	0.2969
PWY0-1415	superpathway of heme biosynthesis from uroporphyrinogen-III	0.2973
PWY-6609	adenine and adenosine salvage III	0.2995
VALDEG-PWY	L-valine degradation I	0.3067
GALACTUROCAT-PWY	D-galacturonate degradation I	0.3074
PWY-6163	chorismate biosynthesis from 3-dehydroquinate	0.3080
PWY-6121	5-aminoimidazole ribonucleotide biosynthesis I	0.3123
DAPLYSINESYN-PWY	L-lysine biosynthesis I	0.3134
PENTOSE-P-PWY	pentose phosphate pathway	0.3159
ECASYN-PWY	enterobacterial common antigen biosynthesis	0.3236
PWY-1541	superpathway of taurine degradation	0.3313
PWY-5198	factor 420 biosynthesis	0.3313
PWY-5743	3-hydroxypropanoate cycle	0.3313
PWY-5744	glyoxylate assimilation	0.3313
PWY-6404	superpathway of mycolyl-arabinogalactan-peptidoglycan complex biosynthesis	0.3313
PWY-6641	superpathway of sulfolactate degradation	0.3313
PWY-6906	chitin derivatives degradation	0.3313
PWY-6948	sitosterol degradation to androstenedione	0.3313
PWY-7024	superpathway of the 3-hydroxypropanoate cycle	0.3313

GLYCOLYSIS-TCA-GLYOX-BYPASS	superpathway of glycolysis, pyruvate dehydrogenase, TCA, and glyoxylate bypass	0.3315
P42-PWY	incomplete reductive TCA cycle	0.3328
DENOVOPURINE2-PWY	superpathway of purine nucleotides de novo biosynthesis II	0.3334
PWY-5304	superpathway of sulfur oxidation (Acidianus ambivalens)	0.3348
PWY-6122	5-aminoimidazole ribonucleotide biosynthesis II	0.3349
PWY-6277	superpathway of 5-aminoimidazole ribonucleotide biosynthesis	0.3349
LIPASYN-PWY	phospholipases	0.3409
P261-PWY	coenzyme M biosynthesis I	0.3409
PWY-2221	Entner-Doudoroff pathway III (semi-phosphorylative)	0.3409
PWY-6174	mevalonate pathway II (archaea)	0.3409
PWY-6992	1,5-anhydrofructose degradation	0.3409
PWY-5676	acetyl-CoA fermentation to butanoate II	0.3469
ARG+POLYAMINE-SYN	superpathway of arginine and polyamine biosynthesis	0.3589
PWY-6545	pyrimidine deoxyribonucleotides de novo biosynthesis III	0.3596
PWY-7228	superpathway of guanosine nucleotides de novo biosynthesis I	0.3613
NONMEVIPP-PWY	methylerythritol phosphate pathway I	0.3641
PWY-7560	methylerythritol phosphate pathway II	0.3641
FUC-RHAMCAT-PWY	superpathway of fucose and rhamnose degradation	0.3650
P23-PWY	reductive TCA cycle I	0.3666
RHAMCAT-PWY	L-rhamnose degradation I	0.3670
POLYAMSYN-PWY	superpathway of polyamine biosynthesis I	0.3681
PWY-6969	TCA cycle V (2-oxoglutarate:ferredoxin oxidoreductase)	0.3712
PWY-5484	glycolysis II (from fructose 6-phosphate)	0.3724
PWY-7255	ergothioneine biosynthesis I (bacteria)	0.3747
PWY-5913	TCA cycle VI (obligate autotrophs)	0.3829
ILEUSYN-PWY	L-isoleucine biosynthesis I (from threonine)	0.3833

VALSYN-PWY	L-valine biosynthesis	0.3833
GLYOXYLATE-BYPASS	glyoxylate cycle	0.3834
TCA	TCA cycle I (prokaryotic)	0.3862
PWY-6471	peptidoglycan biosynthesis IV (Enterococcus faecium)	0.3937
PWY-7196	superpathway of pyrimidine ribonucleosides salvage	0.3971
GLYCOLYSIS-E-D	superpathway of glycolysis and Entner-Doudoroff	0.3997
GLUTORN-PWY	L-ornithine biosynthesis	0.4000
PWY-5989	stearate biosynthesis II (bacteria and plants)	0.4046
TCA-GLYOX-BYPASS	superpathway of glyoxylate bypass and TCA	0.4048
PWY-5860	superpathway of demethylmenaquinol-6 biosynthesis I	0.4066
PWY-5850	superpathway of menaquinol-6 biosynthesis I	0.4067
PWY-5896	superpathway of menaquinol-10 biosynthesis	0.4067
ARGDEG-PWY	superpathway of L-arginine, putrescine, and 4-aminobutanoate degradation	0.4087
ORNARGDEG-PWY	superpathway of L-arginine and L-ornithine degradation	0.4087
GLYCOLYSIS	glycolysis I (from glucose 6-phosphate)	0.4122
PWY0-41	allantoin degradation IV (anaerobic)	0.4144
PWY-2941	L-lysine biosynthesis II	0.4172
PWY-5705	allantoin degradation to glyoxylate III	0.4184
PWY-7211	superpathway of pyrimidine deoxyribonucleotides de novo biosynthesis	0.4188
THREOCAT-PWY	superpathway of L-threonine metabolism	0.4254
PWY-5862	superpathway of demethylmenaquinol-9 biosynthesis	0.4264
P162-PWY	L-glutamate degradation V (via hydroxyglutarate)	0.4265
PWY-5845	superpathway of menaquinol-9 biosynthesis	0.4266
PWY0-862	(5Z)-dodec-5-enoate biosynthesis	0.4272
PWY-5121	superpathway of geranylgeranyl diphosphate biosynthesis II (via MEP)	0.4274
PWY-7187	pyrimidine deoxyribonucleotides de novo biosynthesis II	0.4281

PWY-841	superpathway of purine nucleotides de novo biosynthesis I	0.4302
PWY-5101	L-isoleucine biosynthesis II	0.4344
PWY-7376	cob(II)yrinate a,c-diamide biosynthesis II (late cobalt incorporation)	0.4368
PWY0-1533	methylphosphonate degradation I	0.4385
PWY0-1241	ADP-L-glycero-β-D-manno-heptose biosynthesis	0.4404
DTDPRHAMSYN-PWY	dTDP-L-rhamnose biosynthesis I	0.4407
PWY-7237	myo-, chiro- and scillo-inositol degradation	0.4408
PROTOCATECHUATE-ORTHO-CLEAVAGE-PWY	protocatechuate degradation II (ortho-cleavage pathway)	0.4425
PWY-5420	catechol degradation II (meta-cleavage pathway)	0.4451
PWY-7663	gondoate biosynthesis (anaerobic)	0.4495
RIBOSYN2-PWY	flavin biosynthesis I (bacteria and plants)	0.4520
PWY-7456	mannan degradation	0.4606
LEU-DEG2-PWY	L-leucine degradation I	0.4613
ANAGLYCOLYSIS-PWY	glycolysis III (from glucose)	0.4620
PWY-6123	inosine-5'-phosphate biosynthesis I	0.4621
PWY-6737	starch degradation V	0.4738
PWY-722	nicotinate degradation I	0.4747
PWY0-1338	polymyxin resistance	0.4768
PWY-7007	methyl ketone biosynthesis	0.4799
DHGLUCONATE-PYR-CAT-PWY	glucose degradation (oxidative)	0.4815
AST-PWY	L-arginine degradation II (AST pathway)	0.4824
PWY-7431	aromatic biogenic amine degradation (bacteria)	0.4850
TYRFUMCAT-PWY	L-tyrosine degradation I	0.4858
PWY-5180	toluene degradation I (aerobic) (via o-cresol)	0.4861
PWY-5182	toluene degradation II (aerobic) (via 4-methylcatechol)	0.4861

3-HYDROXYPHENYLACETATE-DEGRADATION-PWY	4-hydroxyphenylacetate degradation	0.4871
PWY-7539	6-hydroxymethyl-dihydropterin diphosphate biosynthesis III (Chlamydia)	0.4875
PWY0-1061	superpathway of L-alanine biosynthesis	0.4916
PWY-7197	pyrimidine deoxyribonucleotide phosphorylation	0.4928
PWY-6700	queuosine biosynthesis	0.4929
PWY-5417	catechol degradation III (ortho-cleavage pathway)	0.4957
PWY-5431	aromatic compounds degradation via β-ketoadipate	0.4957
PWY-7184	pyrimidine deoxyribonucleotides de novo biosynthesis I	0.4964
P381-PWY	adenosylcobalamin biosynthesis II (late cobalt incorporation)	0.4968
SO4ASSIM-PWY	sulfate reduction I (assimilatory)	0.4988
PWY-6507	4-deoxy-L-threo-hex-4-enopyranuronate degradation	0.5010
P221-PWY	octane oxidation	0.5014
CATECHOL-ORTHO-CLEAVAGE-PWY	catechol degradation to β-ketoadipate	0.5035
PWY-7094	fatty acid salvage	0.5053
FAO-PWY	fatty acid β-oxidation I	0.5055
PWY-7377	cob(II)yrinate a,c-diamide biosynthesis I (early cobalt insertion)	0.5056
PWY0-1298	superpathway of pyrimidine deoxyribonucleosides degradation	0.5063
PWY-5028	L-histidine degradation II	0.5077
PWY-5177	glutaryl-CoA degradation	0.5083
PWY-7031	protein N-glycosylation (bacterial)	0.5122
PWY-6588	pyruvate fermentation to acetone	0.5155
PWY-5973	cis-vaccenate biosynthesis	0.5171
P562-PWY	myo-inositol degradation I	0.5171
HCAMHPDEG-PWY	3-phenylpropanoate and 3-(3-hydroxyphenyl)propanoate degradation to 2-oxopent-4-enoate	0.5179

PWY-6690	cinnamate and 3-hydroxycinnamate degradation to 2-oxopent-4-enoate	0.5179
TEICHOICACID-PWY	teichoic acid (poly-glycerol) biosynthesis	0.5204
CALVIN-PWY	Calvin-Benson-Bassham cycle	0.5212
PWY-5659	GDP-mannose biosynthesis	0.5212
PWY-6282	palmitoleate biosynthesis I (from (5Z)-dodec-5-enoate)	0.5217
RUMP-PWY	formaldehyde oxidation I	0.5292
PWY-1861	formaldehyde assimilation II (RuMP Cycle)	0.5301
PPGPPMET-PWY	ppGpp biosynthesis	0.5373
UBISYN-PWY	superpathway of ubiquinol-8 biosynthesis (prokaryotic)	0.5382
PWY-6147	6-hydroxymethyl-dihydropterin diphosphate biosynthesis I	0.5399
PWY-5855	ubiquinol-7 biosynthesis (prokaryotic)	0.5425
PWY-5856	ubiquinol-9 biosynthesis (prokaryotic)	0.5425
PWY-5857	ubiquinol-10 biosynthesis (prokaryotic)	0.5425
PWY-6708	ubiquinol-8 biosynthesis (prokaryotic)	0.5425
ASPASN-PWY	superpathway of L-aspartate and L-asparagine biosynthesis	0.5445
TRPSYN-PWY	L-tryptophan biosynthesis	0.5463
GLUCOSE1PMETAB-PWY	glucose and glucose-1-phosphate degradation	0.5470
PWY-5747	2-methylcitrate cycle II	0.5498
P163-PWY	L-lysine fermentation to acetate and butanoate	0.5538
PWY-7200	superpathway of pyrimidine deoxyribonucleoside salvage	0.5540
ARGSYN-PWY	L-arginine biosynthesis I (via L-ornithine)	0.5554
PWY0-42	2-methylcitrate cycle I	0.5554
PWY-7373	superpathway of demethylmenaquinol-6 biosynthesis II	0.5566
PWY-7371	1,4-dihydroxy-6-naphthoate biosynthesis II	0.5600
PWY-5154	L-arginine biosynthesis III (via N-acetyl-L-citrulline)	0.5606
PWY-7111	pyruvate fermentation to isobutanol (engineered)	0.5626

PWY-7527	L-methionine salvage cycle III	0.5658
PWY-4361	S-methyl-5-thio-α-D-ribose 1-phosphate degradation	0.5667
PWY-5430	meta cleavage pathway of aromatic compounds	0.5668
PWY-6107	chlorosalicylate degradation	0.5677
PWY-7254	TCA cycle VII (acetate-producers)	0.5680
P621-PWY	nylon-6 oligomer degradation	0.5695
P441-PWY	superpathway of N-acetylneuraminate degradation	0.5696
PWY-5384	sucrose degradation IV (sucrose phosphorylase)	0.5706
PWY-7400	L-arginine biosynthesis IV (archaebacteria)	0.5707
PWY-6608	guanosine nucleotides degradation III	0.5712
SER-GLYSYN-PWY	superpathway of L-serine and glycine biosynthesis I	0.5725
PWY-5677	succinate fermentation to butanoate	0.5728
PWY-6263	superpathway of menaquinol-8 biosynthesis II	0.5731
PWY-7664	oleate biosynthesis IV (anaerobic)	0.5750
NAGLIPASYN-PWY	lipid IVA biosynthesis	0.5791
SULFATE-CYS-PWY	superpathway of sulfate assimilation and cysteine biosynthesis	0.5831
NONOXIPENT-PWY	pentose phosphate pathway (non-oxidative branch)	0.5856
ARGSYNBSUB-PWY	L-arginine biosynthesis II (acetyl cycle)	0.5878
PWY-5100	pyruvate fermentation to acetate and lactate II	0.5918
HISTSYN-PWY	L-histidine biosynthesis	0.5945
CENTFERM-PWY	pyruvate fermentation to butanoate	0.5946
PWY490-3	nitrate reduction VI (assimilatory)	0.5953
ARGORNPROST-PWY	arginine, ornithine and proline interconversion	0.5960
PWY-7332	superpathway of UDP-N-acetylglucosamine-derived O-antigen building blocks biosynthesis	0.5978
PWY-6071	superpathway of phenylethylamine degradation	0.6015

PYRIDNUCSYN-PWY	NAD biosynthesis I (from aspartate)	0.6062
PWY-6728	methylaspartate cycle	0.6067
PWY-621	sucrose degradation III (sucrose invertase)	0.6069
PANTO-PWY	phosphopantothenate biosynthesis I	0.6100
PWY-6590	superpathway of Clostridium acetobutylicum acidogenic fermentation	0.6114
GALLATE-DEGRADATION-I-PWY	gallate degradation II	0.6147
METHYLGALLATE-DEGRADATION-PWY	methylgallate degradation	0.6147
PWY-6151	S-adenosyl-L-methionine cycle I	0.6178
HISDEG-PWY	L-histidine degradation I	0.6219
FASYN-ELONG-PWY	fatty acid elongation -- saturated	0.6242
POLYAMINSYN3-PWY	superpathway of polyamine biosynthesis II	0.6254
GALLATE-DEGRADATION-II-PWY	gallate degradation I	0.6268
PWY-6470	peptidoglycan biosynthesis V (β-lactam resistance)	0.6273
PWY-7210	pyrimidine deoxyribonucleotides biosynthesis from CTP	0.6336
PWY-7198	pyrimidine deoxyribonucleotides de novo biosynthesis IV	0.6336
PWY-7199	pyrimidine deoxyribonucleosides salvage	0.6347
P108-PWY	pyruvate fermentation to propanoate I	0.6373
PWY-5103	L-isoleucine biosynthesis III	0.6374
PWY-5507	adenosylcobalamin biosynthesis I (early cobalt insertion)	0.6386
PWY-6628	superpathway of L-phenylalanine biosynthesis	0.6402
PWY4FS-7	phosphatidylglycerol biosynthesis I (plastidic)	0.6433
PWY4FS-8	phosphatidylglycerol biosynthesis II (non-plastidic)	0.6433
FASYN-INITIAL-PWY	superpathway of fatty acid biosynthesis initiation (E. coli)	0.6535
P4-PWY	superpathway of L-lysine, L-threonine and L-methionine biosynthesis I	0.6620
POLYISOPRENSYN-PWY	polyisoprenoid biosynthesis (E. coli)	0.6652
PWY-7328	superpathway of UDP-glucose-derived O-antigen building blocks biosynthesis	0.6682

TRNA-CHARGING-PWY	tRNA charging	0.6818
PWY-181	photorespiration	0.6827
PWY-7391	isoprene biosynthesis II (engineered)	0.6849
HEMESYN2-PWY	heme biosynthesis II (anaerobic)	0.6860
PWY-5345	superpathway of L-methionine biosynthesis (by sulfhydrylation)	0.6881
FOLSYN-PWY	superpathway of tetrahydrofolate biosynthesis and salvage	0.6896
PWY-6338	superpathway of vanillin and vanillate degradation	0.6976
PWY-7097	vanillin and vanillate degradation I	0.6976
PWY-7098	vanillin and vanillate degradation II	0.6976
HEXITOLDEGSUPER-PWY	superpathway of hexitol degradation (bacteria)	0.7012
PWY-6386	UDP-N-acetylmuramoyl-pentapeptide biosynthesis II (lysine-containing)	0.7085
PWY-6612	superpathway of tetrahydrofolate biosynthesis	0.7141
PWY-6339	syringate degradation	0.7164
GLUCUROCAT-PWY	superpathway of β-D-glucuronide and D-glucuronate degradation	0.7225
PWY-6396	superpathway of 2,3-butanediol biosynthesis	0.7230
HOMOSER-METSYN-PWY	L-methionine biosynthesis I	0.7272
GLYCOGENSYNTH-PWY	glycogen biosynthesis I (from ADP-D-Glucose)	0.7274
PWY-6901	superpathway of glucose and xylose degradation	0.7354
PWY-6182	superpathway of salicylate degradation	0.7375
PWY0-321	phenylacetate degradation I (aerobic)	0.7426
1CMET2-PWY	N10-formyl-tetrahydrofolate biosynthesis	0.7429
HEME-BIOSYNTHESIS-II	heme biosynthesis I (aerobic)	0.7456
P184-PWY	protocatechuate degradation I (meta-cleavage pathway)	0.7465
PWY-7003	glycerol degradation to butanol	0.7495
PYRIDNUCSAL-PWY	NAD salvage pathway I	0.7508
BRANCHED-CHAIN-AA-SYN-PWY	superpathway of branched amino acid biosynthesis	0.7508

PWY-7347	sucrose biosynthesis III	0.7526
PEPTIDOGLYCANSYN-PWY	peptidoglycan biosynthesis I (meso-diaminopimelate containing)	0.7528
PWY-6385	peptidoglycan biosynthesis III (mycobacteria)	0.7532
PWY-6876	isopropanol biosynthesis	0.7554
PWY-6387	UDP-N-acetylmuramoyl-pentapeptide biosynthesis I (meso-diaminopimelate containing)	0.7570
PYRIDOXSYN-PWY	pyridoxal 5'-phosphate biosynthesis I	0.7584
SUCSYN-PWY	sucrose biosynthesis I (from photosynthesis)	0.7671
PWY-4984	urea cycle	0.7754
PWY-3941	β-alanine biosynthesis II	0.7782
PWY-2942	L-lysine biosynthesis III	0.7970
PWY-5022	4-aminobutanoate degradation V	0.8015
PWY-5941	glycogen degradation II (eukaryotic)	0.8086
PANTOSYN-PWY	pantothenate and coenzyme A biosynthesis I	0.8127
PWY-4722	creatinine degradation II	0.8159
PWY-5686	UMP biosynthesis	0.8173
PWY-7090	UDP-2,3-diacetamido-2,3-dideoxy-α-D-mannuronate biosynthesis	0.8190
PWY-7323	superpathway of GDP-mannose-derived O-antigen building blocks biosynthesis	0.8275
PWY-7374	1,4-dihydroxy-6-naphthoate biosynthesis I	0.8303
PWY-6897	thiamin salvage II	0.8351
PWY-7446	sulfoglycolysis	0.8374
GLYCOCAT-PWY	glycogen degradation I (bacterial)	0.8381
P281-PWY	3-phenylpropanoate degradation	0.8385
PWY-5654	2-amino-3-carboxymuconate semialdehyde degradation to 2-oxopentenoate	0.8406
PWY-5647	2-nitrobenzoate degradation I	0.8407
PWY-5655	L-tryptophan degradation IX	0.8426

PWY-5695	urate biosynthesis/inosine 5'-phosphate degradation	0.8429
PWY-7221	guanosine ribonucleotides de novo biosynthesis	0.8434
PWY-5651	L-tryptophan degradation to 2-amino-3-carboxymuconate semialdehyde	0.8466
NADSYN-PWY	NAD biosynthesis II (from tryptophan)	0.8471
THRESYN-PWY	superpathway of L-threonine biosynthesis	0.8482
PWY-3001	superpathway of L-isoleucine biosynthesis I	0.8507
PWY-5918	superpathay of heme biosynthesis from glutamate	0.8573
ALL-CHORISMATE-PWY	superpathway of chorismate metabolism	0.8625
COBALSYN-PWY	adenosylcobalamin salvage from cobinamide I	0.8703
HSERMETANA-PWY	L-methionine biosynthesis III	0.8773
PWY-5529	superpathway of bacteriochlorophyll a biosynthesis	0.8813
PWY-5265	peptidoglycan biosynthesis II (staphylococci)	0.8831
PWY-6519	8-amino-7-oxononanoate biosynthesis I	0.8857
PWY-5509	adenosylcobalamin biosynthesis from cobyrinate a,c-diamide I	0.8878
PWY-6269	adenosylcobalamin salvage from cobinamide II	0.8879
MET-SAM-PWY	superpathway of S-adenosyl-L-methionine biosynthesis	0.8895
PWY-6703	preQ0 biosynthesis	0.8913
ANAEROFRUCAT-PWY	homolactic fermentation	0.8927
PWY-7234	inosine-5'-phosphate biosynthesis III	0.8938
PWY-7392	taxadiene biosynthesis (engineered)	0.9076
PWYG-321	mycolate biosynthesis	0.9081
PWY-5347	superpathway of L-methionine biosynthesis (transsulfuration)	0.9124
PWY-7242	D-fructuronate degradation	0.9155
PWY-7616	methanol oxidation to carbon dioxide	0.9160
LACTOSECAT-PWY	lactose and galactose degradation I	0.9162
REDCITCYC	TCA cycle VIII (helicobacter)	0.9177

PWY-6629	superpathway of L-tryptophan biosynthesis	0.9184
LPSSYN-PWY	superpathway of lipopolysaccharide biosynthesis	0.9209
COA-PWY	coenzyme A biosynthesis I	0.9210
PWY-5097	L-lysine biosynthesis VI	0.9219
CHLOROPHYLL-SYN	chlorophyllide a biosynthesis I (aerobic, light-dependent)	0.9236
PWY-1622	formaldehyde assimilation I (serine pathway)	0.9258
PWY-7208	superpathway of pyrimidine nucleobases salvage	0.9363
GALACTARDEG-PWY	D-galactarate degradation I	0.9372
GLUCARGALACTSUPER-PWY	superpathway of D-glucarate and D-galactarate degradation	0.9372
GLUCARDEG-PWY	D-glucarate degradation I	0.9372
PWY-5741	ethylmalonyl-CoA pathway	0.9379
P241-PWY	coenzyme B biosynthesis	0.9423
PWY0-1261	anhydromuropeptides recycling	0.9519
GLUCONEO-PWY	gluconeogenesis I	0.9575
PWY-6478	GDP-D-glycero-α-D-manno-heptose biosynthesis	0.9600
PWY-7219	adenosine ribonucleotides de novo biosynthesis	0.9639
PWY-6467	Kdo transfer to lipid IVA III (Chlamydia)	0.9644
PWY-7229	superpathway of adenosine nucleotides de novo biosynthesis I	0.9685
PWY-5531	chlorophyllide a biosynthesis II (anaerobic)	0.9746
PWY-7159	chlorophyllide a biosynthesis III (aerobic, light independent)	0.9746
PWY-5189	tetrapyrrole biosynthesis II (from glycine)	0.9757
PWY-5505	L-glutamate and L-glutamine biosynthesis	0.9775
PWY-5188	tetrapyrrole biosynthesis I (from glutamate)	0.9777
PWY-1269	CMP-3-deoxy-D-manno-octulosonate biosynthesis I	0.9806
BIOTIN-BIOSYNTHESIS-PWY	biotin biosynthesis I	0.9816
PWY-3781	aerobic respiration I (cytochrome c)	0.9824

GALACT-GLUCUROCAT-PWY	superpathway of hexuronide and hexuronate degradation	0.9845
PWY-7220	adenosine deoxyribonucleotides de novo biosynthesis II	0.9861
PWY-7222	guanosine deoxyribonucleotides de novo biosynthesis II	0.9861
PWY-6630	superpathway of L-tyrosine biosynthesis	0.9886
ORNDEG-PWY	superpathway of ornithine degradation	0.9891
PWY-6126	superpathway of adenosine nucleotides de novo biosynthesis II	0.9928
PWY-6185	4-methylcatechol degradation (ortho cleavage)	0.9960
PWY-5181	toluene degradation III (aerobic) (via p-cresol)	0.9964
AEROBACTINSYN-PWY	aerobactin biosynthesis	1.0000
PWY-1422	vitamin E biosynthesis (tocopherols)	1.0000
PWY-1882	superpathway of C1 compounds oxidation to CO2	1.0000
PWY-3801	sucrose degradation II (sucrose synthase)	1.0000
PWY-7295	L-arabinose degradation IV	1.0000

Bold: P <0.05; * also indicated in comparison between VLS and LA groups

Table S5. The predicted metabolic pathways and the results of statistical tests between VLS and LA group

Pathway	Description	p-values(Welch's t-test)
HEXITOLDEGSUPER-PWY	superpathway of hexitol degradation (bacteria)	0.0022
P461-PWY	hexitol fermentation to lactate, formate, ethanol and acetate	0.0024
FUC-RHAMCAT-PWY	superpathway of fucose and rhamnose degradation	0.0025
COBALSYN-PWY	adenosylcobalamin salvage from cobinamide I	0.0025
PWY-6269	adenosylcobalamin salvage from cobinamide II	0.0027
PWY-5509	adenosylcobalamin biosynthesis from cobyrinate a,c-diamide I	0.0027
GLYCOL-GLYOXDEG-PWY	superpathway of glycol metabolism and degradation	0.0059
PWY-181	photorespiration	0.0060
DENITRIFICATION-PWY	nitrate reduction I (denitrification)	0.0064
**METH-ACETATE-PWY**	**methanogenesis from acetate**	**0.0066***
CODH-PWY	reductive acetyl coenzyme A pathway	0.0085
PWY-5705	allantoin degradation to glyoxylate III	0.0094
PWY-5677	succinate fermentation to butanoate	0.0103
GALACTUROCAT-PWY	D-galacturonate degradation I	0.0112
PWY-6467	Kdo transfer to lipid IVA III (Chlamydia)	0.0112
P441-PWY	superpathway of N-acetylneuraminate degradation	0.0114
P163-PWY	L-lysine fermentation to acetate and butanoate	0.0125
PWY-6588	pyruvate fermentation to acetone	0.0156
GALACT-GLUCUROCAT-PWY	superpathway of hexuronide and hexuronate degradation	0.0162
PWY-5741	ethylmalonyl-CoA pathway	0.0178
PWY-7456	mannan degradation	0.0180
P125-PWY	superpathway of (R,R)-butanediol biosynthesis	0.0182

**GOLPDLCAT-PWY**	**superpathway of glycerol degradation to 1,3-propanediol**	**0.0188***
PWY-1622	formaldehyde assimilation I (serine pathway)	0.0189
PWY490-3	nitrate reduction VI (assimilatory)	0.0189
PWY-1269	CMP-3-deoxy-D-manno-octulosonate biosynthesis I	0.0210
PWY-5180	toluene degradation I (aerobic) (via o-cresol)	0.0215
PWY-5182	toluene degradation II (aerobic) (via 4-methylcatechol)	0.0215
BIOTIN-BIOSYNTHESIS-PWY	biotin biosynthesis I	0.0256
LACTOSECAT-PWY	lactose and galactose degradation I	0.0268
CHLOROPHYLL-SYN	chlorophyllide a biosynthesis I (aerobic, light-dependent)	0.0298
PWY-5531	chlorophyllide a biosynthesis II (anaerobic)	0.0315
PWY-7159	chlorophyllide a biosynthesis III (aerobic, light independent)	0.0315
PWY-5529	superpathway of bacteriochlorophyll a biosynthesis	0.0319
PWY-5676	acetyl-CoA fermentation to butanoate II	0.0349
PWY-6728	methylaspartate cycle	0.0357
PWY-7347	sucrose biosynthesis III	0.0378
PWY-7013	L-1,2-propanediol degradation	0.0384
PWY-6507	4-deoxy-L-threo-hex-4-enopyranuronate degradation	0.0385
SUCSYN-PWY	sucrose biosynthesis I (from photosynthesis)	0.0386
PYRIDOXSYN-PWY	pyridoxal 5'-phosphate biosynthesis I	0.0401
PWY-4361	S-methyl-5-thio-α-D-ribose 1-phosphate degradation	0.0431
PWY-7527	L-methionine salvage cycle III	0.0433
PWY-5088	L-glutamate degradation VIII (to propanoate)	0.0440
CENTFERM-PWY	pyruvate fermentation to butanoate	0.0448
PWY-6590	superpathway of Clostridium acetobutylicum acidogenic fermentation	0.0471
PYRIDNUCSAL-PWY	NAD salvage pathway I	0.0486
PWY-6182	superpathway of salicylate degradation	0.0489

PWY-6892	thiazole biosynthesis I (E. coli)	0.0497
MET-SAM-PWY	superpathway of S-adenosyl-L-methionine biosynthesis	0.0504
PANTOSYN-PWY	pantothenate and coenzyme A biosynthesis I	0.0517
PWY-5198	factor 420 biosynthesis	0.0537
PWY-5304	superpathway of sulfur oxidation (Acidianus ambivalens)	0.0580
PWY-922	mevalonate pathway I	0.0598
PANTO-PWY	phosphopantothenate biosynthesis I	0.0599
PWY-5910	superpathway of geranylgeranyldiphosphate biosynthesis I (via mevalonate)	0.0600
PWY-6628	superpathway of L-phenylalanine biosynthesis	0.0602
PWY0-1298	superpathway of pyrimidine deoxyribonucleosides degradation	0.0617
PWY-7374	1,4-dihydroxy-6-naphthoate biosynthesis I	0.0624
PWY0-1297	superpathway of purine deoxyribonucleosides degradation	0.0656
PWY-1422	vitamin E biosynthesis (tocopherols)	0.0660
PWY-3001	superpathway of L-isoleucine biosynthesis I	0.0698
PWY-5347	superpathway of L-methionine biosynthesis (transsulfuration)	0.0764
P108-PWY	pyruvate fermentation to propanoate I	0.0770
PWY-6519	8-amino-7-oxononanoate biosynthesis I	0.0872
PWY-6731	starch degradation III	0.0896
PWY-3801	sucrose degradation II (sucrose synthase)	0.0906
PWY-6901	superpathway of glucose and xylose degradation	0.1022
P122-PWY	heterolactic fermentation	0.1054
P124-PWY	Bifidobacterium shunt	0.1070
SALVADEHYPOX-PWY	adenosine nucleotides degradation II	0.1171
RHAMCAT-PWY	L-rhamnose degradation I	0.1209
TEICHOICACID-PWY	teichoic acid (poly-glycerol) biosynthesis	0.1249
PWY-6572	chondroitin sulfate degradation I (bacterial)	0.1252

THISYN-PWY	superpathway of thiamin diphosphate biosynthesis I	0.1374
GLYCOLYSIS-TCA-GLYOX-BYPASS	superpathway of glycolysis, pyruvate dehydrogenase, TCA, and glyoxylate bypass	0.1434
P621-PWY	nylon-6 oligomer degradation	0.1445
RUMP-PWY	formaldehyde oxidation I	0.1466
PWY-1861	formaldehyde assimilation II (RuMP Cycle)	0.1478
PWY-5419	catechol degradation to 2-oxopent-4-enoate II	0.1492
PWY-5845	superpathway of menaquinol-9 biosynthesis	0.1510
PWY-5862	superpathway of demethylmenaquinol-9 biosynthesis	0.1532
PWY-6107	chlorosalicylate degradation	0.1555
PWY-5850	superpathway of menaquinol-6 biosynthesis I	0.1557
PWY-5896	superpathway of menaquinol-10 biosynthesis	0.1557
PWY-5860	superpathway of demethylmenaquinol-6 biosynthesis I	0.1577
THRESYN-PWY	superpathway of L-threonine biosynthesis	0.1593
OANTIGEN-PWY	O-antigen building blocks biosynthesis (E. coli)	0.1618
P261-PWY	coenzyme M biosynthesis I	0.1648
PWY-5420	catechol degradation II (meta-cleavage pathway)	0.1705
P101-PWY	ectoine biosynthesis	0.1713
PWY0-1479	tRNA processing	0.1754
1CMET2-PWY	N10-formyl-tetrahydrofolate biosynthesis	0.1821
PWY-7031	protein N-glycosylation (bacterial)	0.1865
PWY-7373	superpathway of demethylmenaquinol-6 biosynthesis II	0.1871
PWY-7371	1,4-dihydroxy-6-naphthoate biosynthesis II	0.1878
PWY-6713	L-rhamnose degradation II	0.1881
P105-PWY	TCA cycle IV (2-oxoglutarate decarboxylase)	0.1899
PWY-6749	CMP-legionaminate biosynthesis I	0.1935
PWY-6263	superpathway of menaquinol-8 biosynthesis II	0.1968

PWY-2942	L-lysine biosynthesis III	0.1977
PWY-6210	2-aminophenol degradation	0.2004
PWY-6562	norspermidine biosynthesis	0.2014
P162-PWY	L-glutamate degradation V (via hydroxyglutarate)	0.2033
PWY-6895	superpathway of thiamin diphosphate biosynthesis II	0.2046
PWY-5655	L-tryptophan degradation IX	0.2101
PWY-5647	2-nitrobenzoate degradation I	0.2114
PWY0-1296	purine ribonucleosides degradation	0.2130
PWY-6505	L-tryptophan degradation XII (Geobacillus)	0.2135
PWY-6876	isopropanol biosynthesis	0.2203
ECASYN-PWY	enterobacterial common antigen biosynthesis	0.2244
P241-PWY	coenzyme B biosynthesis	0.2300
PWY-7315	dTDP-N-acetylthomosamine biosynthesis	0.2321
PWY-6608	guanosine nucleotides degradation III	0.2328
PWY-5505	L-glutamate and L-glutamine biosynthesis	0.2353
PWY-7242	D-fructuronate degradation	0.2390
PWY-6125	superpathway of guanosine nucleotides de novo biosynthesis II	0.2409
PWY-6891	thiazole biosynthesis II (Bacillus)	0.2418
HCAMHPDEG-PWY	3-phenylpropanoate and 3-(3-hydroxyphenyl)propanoate degradation to 2-oxopent-4-enoate	0.2444
PWY-6690	cinnamate and 3-hydroxycinnamate degradation to 2-oxopent-4-enoate	0.2444
PWY-6397	mycolyl-arabinogalactan-peptidoglycan complex biosynthesis	0.2475
P281-PWY	3-phenylpropanoate degradation	0.2497
PWY-5097	L-lysine biosynthesis VI	0.2519
COMPLETE-ARO-PWY	superpathway of aromatic amino acid biosynthesis	0.2589
KDO-NAGLIPASYN-PWY	superpathway of (Kdo)2-lipid A biosynthesis	0.2600

PWY1G-0	mycothiol biosynthesis	0.2744
PWY-7332	superpathway of UDP-N-acetylglucosamine-derived O-antigen building blocks biosynthesis	0.2745
PWY-3781	aerobic respiration I (cytochrome c)	0.2856
TRPSYN-PWY	L-tryptophan biosynthesis	0.2928
PWY-7198	pyrimidine deoxyribonucleotides de novo biosynthesis IV	0.2933
PWY-7210	pyrimidine deoxyribonucleotides biosynthesis from CTP	0.2933
NAD-BIOSYNTHESIS-II	NAD salvage pathway II	0.2962
BRANCHED-CHAIN-AA-SYN-PWY	superpathway of branched amino acid biosynthesis	0.2999
ASPASN-PWY	superpathway of L-aspartate and L-asparagine biosynthesis	0.3169
HSERMETANA-PWY	L-methionine biosynthesis III	0.3218
PWY-7391	isoprene biosynthesis II (engineered)	0.3235
P184-PWY	protocatechuate degradation I (meta-cleavage pathway)	0.3313
PWY-1541	superpathway of taurine degradation	0.3313
PWY-3941	β-alanine biosynthesis II	0.3313
PWY-5743	3-hydroxypropanoate cycle	0.3313
PWY-5744	glyoxylate assimilation	0.3313
PWY-6338	superpathway of vanillin and vanillate degradation	0.3313
PWY-6339	syringate degradation	0.3313
PWY-6404	superpathway of mycolyl-arabinogalactan-peptidoglycan complex biosynthesis	0.3313
PWY-6641	superpathway of sulfolactate degradation	0.3313
PWY-6948	sitosterol degradation to androstenedione	0.3313
PWY-7024	superpathway of the 3-hydroxypropanoate cycle	0.3313
PWY-7097	vanillin and vanillate degradation I	0.3313
PWY-7098	vanillin and vanillate degradation II	0.3313
PWY-5920	superpathway of heme biosynthesis from glycine	0.3316

HOMOSER-METSYN-PWY	L-methionine biosynthesis I	0.3323
PWY0-162	superpathway of pyrimidine ribonucleotides de novo biosynthesis	0.3326
AEROBACTINSYN-PWY	aerobactin biosynthesis	0.3343
PWY-1882	superpathway of C1 compounds oxidation to CO2	0.3343
PWY-6992	1,5-anhydrofructose degradation	0.3343
PWY-7295	L-arabinose degradation IV	0.3343
P161-PWY	acetylene degradation	0.3344
ANAEROFRUCAT-PWY	homolactic fermentation	0.3373
PWY-6737	starch degradation V	0.3409
GLYCOLYSIS-E-D	superpathway of glycolysis and Entner-Doudoroff	0.3446
PWY0-1277	3-phenylpropanoate and 3-(3-hydroxyphenyl)propanoate degradation	0.3521
PWY-7090	UDP-2,3-diacetamido-2,3-dideoxy-α-D-mannuronate biosynthesis	0.3532
PHOSLIPSYN-PWY	superpathway of phospholipid biosynthesis I (bacteria)	0.3557
PWY-2941	L-lysine biosynthesis II	0.3614
PWY-6071	superpathway of phenylethylamine degradation	0.3622
PWY-6163	chorismate biosynthesis from 3-dehydroquinate	0.3759
PWY-7255	ergothioneine biosynthesis I (bacteria)	0.3782
PWY-6185	4-methylcatechol degradation (ortho cleavage)	0.3831
ARO-PWY	chorismate biosynthesis I	0.3834
PWY-5181	toluene degradation III (aerobic) (via p-cresol)	0.3835
PWY0-1415	superpathway of heme biosynthesis from uroporphyrinogen-III	0.3924
PWY-6944	androstenedione degradation	0.3969
P23-PWY	reductive TCA cycle I	0.4045
PWY-6478	GDP-D-glycero-α-D-manno-heptose biosynthesis	0.4048
PWY-6630	superpathway of L-tyrosine biosynthesis	0.4053
PWY0-41	allantoin degradation IV (anaerobic)	0.4064

PWY-7616	methanol oxidation to carbon dioxide	0.4110
PWY-7663	gondoate biosynthesis (anaerobic)	0.4160
CRNFORCAT-PWY	creatinine degradation I	0.4167
PWY-6151	S-adenosyl-L-methionine cycle I	0.4173
PWY-7220	adenosine deoxyribonucleotides de novo biosynthesis II	0.4177
PWY-7222	guanosine deoxyribonucleotides de novo biosynthesis II	0.4177
PWY-6897	thiamin salvage II	0.4346
PWY-3661	glycine betaine degradation I	0.4359
PWY0-845	superpathway of pyridoxal 5'-phosphate biosynthesis and salvage	0.4412
CALVIN-PWY	Calvin-Benson-Bassham cycle	0.4473
PWY-7376	cob(II)yrinate a,c-diamide biosynthesis II (late cobalt incorporation)	0.4512
PWY-6147	6-hydroxymethyl-dihydropterin diphosphate biosynthesis I	0.4567
GALLATE-DEGRADATION-II-PWY	gallate degradation I	0.4616
PWY-6969	TCA cycle V (2-oxoglutarate:ferredoxin oxidoreductase)	0.4652
PWY-5430	meta cleavage pathway of aromatic compounds	0.4655
PWY-6126	superpathway of adenosine nucleotides de novo biosynthesis II	0.4747
ALL-CHORISMATE-PWY	superpathway of chorismate metabolism	0.4819
PWY-841	superpathway of purine nucleotides de novo biosynthesis I	0.4900
PWY-7229	superpathway of adenosine nucleotides de novo biosynthesis I	0.5014
PWY-6906	chitin derivatives degradation	0.5019
PWY-6470	peptidoglycan biosynthesis V (β-lactam resistance)	0.5067
PWY-7539	6-hydroxymethyl-dihydropterin diphosphate biosynthesis III (Chlamydia)	0.5105
PWY-6629	superpathway of L-tryptophan biosynthesis	0.5285
PWY0-166	superpathway of pyrimidine deoxyribonucleotides de novo biosynthesis (E. coli)	0.5288
PWY-6353	purine nucleotides degradation II (aerobic)	0.5318
PWY-5667	CDP-diacylglycerol biosynthesis I	0.5406

PWY0-1319	CDP-diacylglycerol biosynthesis II	0.5406
GLUCUROCAT-PWY	superpathway of β-D-glucuronide and D-glucuronate degradation	0.5426
P4-PWY	superpathway of L-lysine, L-threonine and L-methionine biosynthesis I	0.5439
PWY-7446	sulfoglycolysis	0.5492
NAGLIPASYN-PWY	lipid IVA biosynthesis	0.5510
PWY0-321	phenylacetate degradation I (aerobic)	0.5512
PWY-5484	glycolysis II (from fructose 6-phosphate)	0.5538
PWY-5837	1,4-dihydroxy-2-naphthoate biosynthesis I	0.5543
PWY-5659	GDP-mannose biosynthesis	0.5544
PWY-5863	superpathway of phylloquinol biosynthesis	0.5588
GALLATE-DEGRADATION-I-PWY	gallate degradation II	0.5619
METHYLGALLATE-DEGRADATION-PWY	methylgallate degradation	0.5619
PWY-7219	adenosine ribonucleotides de novo biosynthesis	0.5789
PWY0-781	aspartate superpathway	0.5880
UDPNAGSYN-PWY	UDP-N-acetyl-D-glucosamine biosynthesis I	0.5899
GLUCONEO-PWY	gluconeogenesis I	0.5930
PWY0-1586	peptidoglycan maturation (meso-diaminopimelate containing)	0.5960
POLYAMINSYN3-PWY	superpathway of polyamine biosynthesis II	0.5969
PWY-5973	cis-vaccenate biosynthesis	0.6033
KETOGLUCONMET-PWY	ketogluconate metabolism	0.6076
HISTSYN-PWY	L-histidine biosynthesis	0.6097
PWY-6396	superpathway of 2,3-butanediol biosynthesis	0.6196
PWY0-1261	anhydromuropeptides recycling	0.6209
PWY-5941	glycogen degradation II (eukaryotic)	0.6226
PWY-5154	L-arginine biosynthesis III (via N-acetyl-L-citrulline)	0.6288
PWY-7111	pyruvate fermentation to isobutanol (engineered)	0.6294

PWY-6123	inosine-5'-phosphate biosynthesis I	0.6309
PWY-6143	CMP-pseudaminate biosynthesis	0.6337
PWY-7377	cob(II)yrinate a,c-diamide biosynthesis I (early cobalt insertion)	0.6348
PWY-7187	pyrimidine deoxyribonucleotides de novo biosynthesis II	0.6484
HEMESYN2-PWY	heme biosynthesis II (anaerobic)	0.6515
PWY4FS-7	phosphatidylglycerol biosynthesis I (plastidic)	0.6556
PWY4FS-8	phosphatidylglycerol biosynthesis II (non-plastidic)	0.6556
HISDEG-PWY	L-histidine degradation I	0.6632
PWY-6471	peptidoglycan biosynthesis IV (Enterococcus faecium)	0.6646
ENTBACSYN-PWY	enterobactin biosynthesis	0.6726
PWY-5913	TCA cycle VI (obligate autotrophs)	0.6727
PWY0-1241	ADP-L-glycero-β-D-manno-heptose biosynthesis	0.6760
PWY-5022	4-aminobutanoate degradation V	0.6832
GLYCOCAT-PWY	glycogen degradation I (bacterial)	0.6863
NONMEVIPP-PWY	methylerythritol phosphate pathway I	0.6869
PWY-7560	methylerythritol phosphate pathway II	0.6869
PWY-7392	taxadiene biosynthesis (engineered)	0.6889
COLANSYN-PWY	colanic acid building blocks biosynthesis	0.6950
PWY-7323	superpathway of GDP-mannose-derived O-antigen building blocks biosynthesis	0.6953
NONOXIPENT-PWY	pentose phosphate pathway (non-oxidative branch)	0.6963
THREOCAT-PWY	superpathway of L-threonine metabolism	0.7067
GLYCOLYSIS	glycolysis I (from glucose 6-phosphate)	0.7098
PWY-7400	L-arginine biosynthesis IV (archaebacteria)	0.7112
PWY-5178	toluene degradation IV (aerobic) (via catechol)	0.7242
PWY-6317	galactose degradation I (Leloir pathway)	0.7253
FASYN-INITIAL-PWY	superpathway of fatty acid biosynthesis initiation (E. coli)	0.7255

COA-PWY	coenzyme A biosynthesis I	0.7262
DENOVOPURINE2-PWY	superpathway of purine nucleotides de novo biosynthesis II	0.7293
GALACTARDEG-PWY	D-galactarate degradation I	0.7336
GLUCARGALACTSUPER-PWY	superpathway of D-glucarate and D-galactarate degradation	0.7336
GLUCARDEG-PWY	D-glucarate degradation I	0.7336
REDCITCYC	TCA cycle VIII (helicobacter)	0.7366
PWY-5104	L-isoleucine biosynthesis IV	0.7381
VALDEG-PWY	L-valine degradation I	0.7387
ARGSYN-PWY	L-arginine biosynthesis I (via L-ornithine)	0.7398
PWY-7237	myo-, chiro- and scillo-inositol degradation	0.7436
PWY-5121	superpathway of geranylgeranyl diphosphate biosynthesis II (via MEP)	0.7443
P562-PWY	myo-inositol degradation I	0.7489
PWY-621	sucrose degradation III (sucrose invertase)	0.7528
PENTOSE-P-PWY	pentose phosphate pathway	0.7535
PWY-7228	superpathway of guanosine nucleotides de novo biosynthesis I	0.7577
DTDPRHAMSYN-PWY	dTDP-L-rhamnose biosynthesis I	0.7597
PWY-5897	superpathway of menaquinol-11 biosynthesis	0.7643
PWY-5898	superpathway of menaquinol-12 biosynthesis	0.7643
PWY-5899	superpathway of menaquinol-13 biosynthesis	0.7643
PWY-5654	2-amino-3-carboxymuconate semialdehyde degradation to 2-oxopentenoate	0.7654
PWY-5103	L-isoleucine biosynthesis III	0.7658
LPSSYN-PWY	superpathway of lipopolysaccharide biosynthesis	0.7664
PWY-5861	superpathway of demethylmenaquinol-8 biosynthesis	0.7669
TCA	TCA cycle I (prokaryotic)	0.7672
PWY-5651	L-tryptophan degradation to 2-amino-3-carboxymuconate semialdehyde	0.7694
NADSYN-PWY	NAD biosynthesis II (from tryptophan)	0.7695

PWY-5265	peptidoglycan biosynthesis II (staphylococci)	0.7731
GLUTORN-PWY	L-ornithine biosynthesis	0.7763
ORNDEG-PWY	superpathway of ornithine degradation	0.7769
PPGPPMET-PWY	ppGpp biosynthesis	0.7865
PWY-4722	creatinine degradation II	0.7868
PWY-6703	preQ0 biosynthesis	0.7897
PWY-5188	tetrapyrrole biosynthesis I (from glutamate)	0.7899
PWY-5838	superpathway of menaquinol-8 biosynthesis I	0.7941
GLCMANNANAUT-PWY	superpathway of N-acetylglucosamine, N-acetylmannosamine and N-acetylneuraminate degradation	0.7976
PWY-5415	catechol degradation I (meta-cleavage pathway)	0.8035
PWY-5345	superpathway of L-methionine biosynthesis (by sulfhydrylation)	0.8038
PWY0-1338	polymyxin resistance	0.8050
GLYCOGENSYNTH-PWY	glycogen biosynthesis I (from ADP-D-Glucose)	0.8064
PWY-6700	queuosine biosynthesis	0.8065
TCA-GLYOX-BYPASS	superpathway of glyoxylate bypass and TCA	0.8074
PWY-5989	stearate biosynthesis II (bacteria and plants)	0.8088
P381-PWY	adenosylcobalamin biosynthesis II (late cobalt incorporation)	0.8105
PWY-5971	palmitate biosynthesis II (bacteria and plants)	0.8112
GLYOXYLATE-BYPASS	glyoxylate cycle	0.8159
PWY-5384	sucrose degradation IV (sucrose phosphorylase)	0.8209
PWY-7197	pyrimidine deoxyribonucleotide phosphorylation	0.8222
PWY-5189	tetrapyrrole biosynthesis II (from glycine)	0.8242
PYRIDNUCSYN-PWY	NAD biosynthesis I (from aspartate)	0.8269
PWY-7328	superpathway of UDP-glucose-derived O-antigen building blocks biosynthesis	0.8277
ARGSYNBSUB-PWY	L-arginine biosynthesis II (acetyl cycle)	0.8287

PWY-5101	L-isoleucine biosynthesis II	0.8305
METHGLYUT-PWY	superpathway of methylglyoxal degradation	0.8342
PWY-5005	biotin biosynthesis II	0.8347
FASYN-ELONG-PWY	fatty acid elongation -- saturated	0.8348
FUCCAT-PWY	fucose degradation	0.8367
ARGDEG-PWY	superpathway of L-arginine, putrescine, and 4-aminobutanoate degradation	0.8397
ORNARGDEG-PWY	superpathway of L-arginine and L-ornithine degradation	0.8397
PWY-5507	adenosylcobalamin biosynthesis I (early cobalt insertion)	0.8414
PWY-5177	glutaryl-CoA degradation	0.8423
PWY-6383	mono-trans, poly-cis decaprenyl phosphate biosynthesis	0.8424
FOLSYN-PWY	superpathway of tetrahydrofolate biosynthesis and salvage	0.8433
PWY-5840	superpathway of menaquinol-7 biosynthesis	0.8469
PRPP-PWY	superpathway of histidine, purine, and pyrimidine biosynthesis	0.8533
PWY-7234	inosine-5'-phosphate biosynthesis III	0.8543
PWY-7184	pyrimidine deoxyribonucleotides de novo biosynthesis I	0.8548
PWY-6282	palmitoleate biosynthesis I (from (5Z)-dodec-5-enoate)	0.8557
PWY-7199	pyrimidine deoxyribonucleosides salvage	0.8591
PWY0-862	(5Z)-dodec-5-enoate biosynthesis	0.8624
PWY-7221	guanosine ribonucleotides de novo biosynthesis	0.8630
PWY-4984	urea cycle	0.8699
PWY0-1533	methylphosphonate degradation I	0.8757
PWY-5918	superpathay of heme biosynthesis from glutamate	0.8759
TRNA-CHARGING-PWY	tRNA charging	0.8775
PWY-722	nicotinate degradation I	0.8824
PWY-5686	UMP biosynthesis	0.8824
ANAGLYCOLYSIS-PWY	glycolysis III (from glucose)	0.8880

FAO-PWY	fatty acid β-oxidation I	0.8891
ILEUSYN-PWY	L-isoleucine biosynthesis I (from threonine)	0.8899
VALSYN-PWY	L-valine biosynthesis	0.8899
SER-GLYSYN-PWY	superpathway of L-serine and glycine biosynthesis I	0.8931
PWY0-1061	superpathway of L-alanine biosynthesis	0.8936
PWY-7007	methyl ketone biosynthesis	0.8979
PWY-7094	fatty acid salvage	0.8979
PWY-6612	superpathway of tetrahydrofolate biosynthesis	0.9001
PROTOCATECHUATE-ORTHO-CLEAVAGE-PWY	protocatechuate degradation II (ortho-cleavage pathway)	0.9030
SO4ASSIM-PWY	sulfate reduction I (assimilatory)	0.9057
POLYISOPRENSYN-PWY	polyisoprenoid biosynthesis (E. coli)	0.9070
PWY-7208	superpathway of pyrimidine nucleobases salvage	0.9070
AST-PWY	L-arginine degradation II (AST pathway)	0.9095
PWY-5747	2-methylcitrate cycle II	0.9116
3-HYDROXYPHENYLACETATE-DEGRADATION-PWY	4-hydroxyphenylacetate degradation	0.9130
TYRFUMCAT-PWY	L-tyrosine degradation I	0.9181
PWY0-42	2-methylcitrate cycle I	0.9208
PWY-7003	glycerol degradation to butanol	0.9213
DHGLUCONATE-PYR-CAT-PWY	glucose degradation (oxidative)	0.9230
P164-PWY	purine nucleobases degradation I (anaerobic)	0.9247
PWY-7200	superpathway of pyrimidine deoxyribonucleoside salvage	0.9259
PWY-6122	5-aminoimidazole ribonucleotide biosynthesis II	0.9267
PWY-6277	superpathway of 5-aminoimidazole ribonucleotide biosynthesis	0.9267
P42-PWY	incomplete reductive TCA cycle	0.9285

PWY-5417	catechol degradation III (ortho-cleavage pathway)	0.9304
PWY-5431	aromatic compounds degradation via β-ketoadipate	0.9304
PWY-6121	5-aminoimidazole ribonucleotide biosynthesis I	0.9306
P221-PWY	octane oxidation	0.9310
ARG+POLYAMINE-SYN	superpathway of arginine and polyamine biosynthesis	0.9314
PWY-5695	urate biosynthesis/inosine 5'-phosphate degradation	0.9322
UBISYN-PWY	superpathway of ubiquinol-8 biosynthesis (prokaryotic)	0.9334
PWY-5100	pyruvate fermentation to acetate and lactate II	0.9335
PWY-5855	ubiquinol-7 biosynthesis (prokaryotic)	0.9407
PWY-5856	ubiquinol-9 biosynthesis (prokaryotic)	0.9407
PWY-5857	ubiquinol-10 biosynthesis (prokaryotic)	0.9407
PWY-6708	ubiquinol-8 biosynthesis (prokaryotic)	0.9407
PWY-7431	aromatic biogenic amine degradation (bacteria)	0.9408
CATECHOL-ORTHO-CLEAVAGE-PWY	catechol degradation to β-ketoadipate	0.9435
PWY-7664	oleate biosynthesis IV (anaerobic)	0.9460
PWY-5028	L-histidine degradation II	0.9468
PWY-7196	superpathway of pyrimidine ribonucleosides salvage	0.9471
PWY-7211	superpathway of pyrimidine deoxyribonucleotides de novo biosynthesis	0.9484
SULFATE-CYS-PWY	superpathway of sulfate assimilation and cysteine biosynthesis	0.9524
PWY-6386	UDP-N-acetylmuramoyl-pentapeptide biosynthesis II (lysine-containing)	0.9542
HEME-BIOSYNTHESIS-II	heme biosynthesis I (aerobic)	0.9544
PWY-6387	UDP-N-acetylmuramoyl-pentapeptide biosynthesis I (meso-diaminopimelate containing)	0.9575
PWY-6609	adenine and adenosine salvage III	0.9620
PWYG-321	mycolate biosynthesis	0.9646
FERMENTATION-PWY	mixed acid fermentation	0.9667

PWY-6545	pyrimidine deoxyribonucleotides de novo biosynthesis III	0.9680
LEU-DEG2-PWY	L-leucine degradation I	0.9790
GLUCOSE1PMETAB-PWY	glucose and glucose-1-phosphate degradation	0.9837
DAPLYSINESYN-PWY	L-lysine biosynthesis I	0.9881
PEPTIDOGLYCANSYN-PWY	peptidoglycan biosynthesis I (meso-diaminopimelate containing)	0.9888
ARGORNPROST-PWY	arginine, ornithine and proline interconversion	0.9920
RIBOSYN2-PWY	flavin biosynthesis I (bacteria and plants)	0.9942
POLYAMSYN-PWY	superpathway of polyamine biosynthesis I	0.9946
PWY-6385	peptidoglycan biosynthesis III (mycobacteria)	0.9949
PWY-7254	TCA cycle VII (acetate-producers)	0.9966
LIPASYN-PWY	phospholipases	1.0000
PWY-2221	Entner-Doudoroff pathway III (semi-phosphorylative)	1.0000
PWY-6174	mevalonate pathway II (archaea)	1.0000

Bold: P <0.05; * also indicated in comparison between VLS and control (NV) groups
